# Dynamics of integron structures across a wastewater network – Implications to resistance gene transfer

**DOI:** 10.1016/j.watres.2021.117720

**Published:** 2021-11-01

**Authors:** Marcos Quintela-Baluja, Dominic Frigon, M. Abouelnaga, Kelly Jobling, Jesús L. Romalde, Mariano Gomez Lopez, David W. Graham

**Affiliations:** a School of Engineering, Cassie Building, Newcastle University, Newcastle upon Tyne NE1 7RU, UK; b Department of Analytical Chemistry, Nutrition and Bromatology, University of Santiago de Compostela, Spain; c Department of Civil Engineering and Applied Mechanics, McGill University, Montréal (QC), Canada; d Department of Analytical Chemistry, School of Veterinary Sciences, Suez Canel University, Ismailia, Egypt; e Departamento de Microbiología y Parasitología, CIBUS-Facultad de Biología & Institute CRETUS, Universidade de Santiago de Compostela, Santiago de Compostela, Spain; f Labaqua, Santiago de Compostela, Spain

**Keywords:** Integron dynamics, Wastewater networks, Class 1 integrons, Empty integron structures, Antibiotic resistance genes, qPCR probes

## Abstract

Class 1 and other integrons are common in wastewater networks, often being associated with antibiotic resistance genes (ARGs). However, the importance of different integron structures in ARG transfer within wastewater systems has only been implied, especially between community and hospital sources, among wastewater treatment plant compartments, and in receiving waters. This uncertainty is partly because current clinical class 1 integron qPCR assays (i.e., that target human-impacted structures, i.e., *clintI1*) poorly delineate *clintI1* from non-impacted class 1 integron structures. They also say nothing about their ARG content. To fill these technical gaps, new real-time qPCR assays were developed for “impacted” class 1 structures (called *aint1*; i.e., anthropogenic class 1 integrons) and empty *aint1* structures (i.e., carry no ARGs; called *eaint1*). The new assays and other integron assays then were used to examine integron dynamics across a wastewater network. 16S metagenomic sequencing also was performed to characterise associated microbiomes. *aint1* abundances per bacterial cell were about 10 times greater in hospital wastewaters compared with other compartments, suggesting *aint1* enrichment with ARGs in hospital sources. Conversely, the relative abundance of *eaint1* structures were over double in recycled activated sludge compared with other compartments, except receiving waters (RAS; ∼30% of RAS class 1 structures did not carry ARGs). Microbiome analysis showed that human-associated bacterial taxa with mobile integrons also differed in RAS and river sediments. Further, class 1 integrons in RAS bacteria appear to have released ARGs, whereas hospital bacteria have accumulated ARGs. Results show that quantifying integron dynamics can help explain where ARG transfer occurs in wastewater networks, and should be considered in future studies on antibiotic resistance in the environment.

## Highlights

• Prevalence and types of integrons vary widely across a wastewater network.
• Human-impacted class 1 integrons carrying ARGs dominate, but are highest in hospital wastewater.
• New qPCR assays are reported that segregate "anthropogenic" class 1 integrons that carry ARGs (aint1) vs empty structures (eaint1).
• Recycled activated sludge has triple the “empty structures” per integron relative to WWTP liquid effluents.
• Integron dynamics help identify wastewater compartments with elevated ARG transfer.

## Graphical abstract

**Figure:**
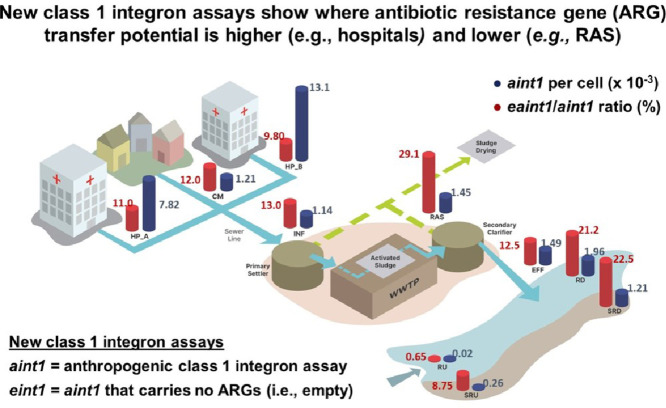

## 1 Introduction

Increasing antibiotic resistance is an evolutionary response to the overuse of antibiotics and environmental pollution, and is a global health concern (Chen and Zhang, 2013; Devarajan et al., 2016; Quintela-Baluja et al., 2015; Xu et al., 2016). Antibiotic use selects for resistance strains in human and animal wastes, which can be released to the environment via wastewater, spreading antibiotic resistance genes (ARGs) and bacteria across nature. Selection is often through versatile gene transfer mechanisms, which allow relatively unconstrained ARG migration across phylogenetic, ecological, and geographic borders. As such, the use of antibiotics in one habitat (e.g., hospitals) can impact resistomes in other locations across the environment. Therefore, understanding the vectors and transfer mechanisms by which bacteria share and acquire ARGs is important, maybe more important than studying the levels of ARGs themselves.

A key group of ARG transmission vectors in bacteria are integrons that are carried on mobile genetic elements (MGEs), including plasmids and transposons (Cambray et al., 2010). Integrons are genetically mobile bacterial recombination systems (i.e., mobile integrons) that allow the acquisition and expression of promoterless protein-coding sequences (gene cassettes; GC). They can confer rapid adaptation and selective advantage to host bacteria (often under specific environmental pressures). A large array of GCs that carry ARGs have been found in integrons associated with MGEs (Cambray et al., 2010; Partridge et al. 2009), which are very diverse and associated with ARG transmission (Cambray et al., 2011).

Three classes of mobile integrons, i.e., class 1, 2, and 3, appear to be most important for acquiring and transmitting ARGs (Cambray et al., 2010). Class 1 integrons (*intI1*) are the most prevalent class among clinical and environmental isolates (Cambray et al., 2010; Cambray et al., 2011; Deng et al., 2015; Partridge et al. 2009). However, all three integron classes (*intI1, intI2*, and *intI3*) are found in human, animal, and environmental metagenomes (Cambray et al., 2010; Deng et al., 2015; Simo Tchuinte et al., 2016; Stokes and Gillings, 2011). These integrons are often associated with *Proteobacteria* species, although *intI1* has also been found in Gram-positive strains (Cambray et al., 2010; Deng et al., 2015; Partridge et al. 2009). Over 130 GCs with ARGs have been found on mobile integrons (Stalder et al., 2012).

Class 1 integrons that carry ARGs are found in both pathogenic and non-pathogenic strains, although not all class 1 integrons are the same. A sub-group of class 1 integrons have been related with “clinical” settings (hereafter called clinical class 1 integrons, “*clintI1*”) (Gillings et al., 2008). *clintI1* differs from other class 1 integrons in that its integron-integrase gene is conserved (5’CS end), but it carries an additional segment at its 3’CS end, which is composed of *qacEΔ1* (low-level resistance to quaternary ammonium compounds), *sul1* (sulfonamide resistance), and *orf5* (unknown function) (Gillings et al., 2008). Like other class 1 integrons, *clintI1* is co-regulated by SOS response systems, suggesting reordering and acquiring existing/exogenous cassettes may be activated under stressed conditions when genetic adaptation is needed (Barraud and Ploy, 2015; Cambray et al., 2011). However, integron regulation is not fully understood - in particular how it influences the dynamics of ARG transmission and antibiotic resistance spread. Understanding the role of integrons within a system is further confused by the fact that many integrons, including *clintI1*, can be found “empty”; e.g., carrying no ARGs (Chakraborty et al. 2013; Chang et al., 2007; Dillon et al., 2005; Moura et al., 2012; Sá et al., 2010; Toval et al., 2015). The incorporation of ARGs into *clintI1* also occurs in the absence of antibiotics (Pérez-Valdespino et al., 2016), suggesting integron and ARG ecology are not solely driven by antibiotic use.

**Fig. 1:**
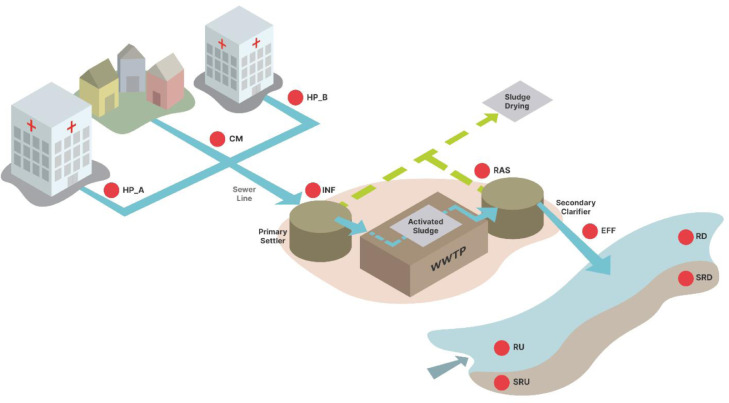
Schematic of sampling points in study area. CM = community wastewater; HP_A, HP_B = wastewater from two different hospitals; INF = wastewater treatment plant (WWTP) influent; EFF = WWTP effluent; RAS = recycled activated sludge; RU= upstream river water column; SRU = upstream river sediment; RD = downstream river water column; and SRD = downstream river sediment. Adapted from Quintela-Baluja et al. (2019).

Here we examine integron dynamics by quantifying different integrons and microbiota across a wastewater network that includes community and hospital sources, sewer lines, a wastewater treatment plant (WWTP), and upstream and downstream receiving waters (Fig. 1). Integron types, states, and possible bacterial hosts (called here “potential bacteria carrying mobile integrons” or PBCMI) were compared across compartments. The goal was to delineate the spatial dynamics of integrons, especially human-impacted class 1 integrons, to help explain where ARG transmission really occurs within wastewater systems. However, to do this, we needed to design new probe-primer sets that better segregate *clintI1* from *intI1*, and probes for quantifying class 1 structures that do not carry ARGs.

## 2 Materials and methods

### 2.1 Study area and sampling procedure

Sampling was performed during the summer in a city in northern Spain, which has limited heavy industry or agriculture. A schematic of the network is shown in Fig. 1. Samples were collected over three weeks, including effluents from two hospitals (HP_A and HP_B); the parallel community sewer line (CM); the WWTP influent (INF) and effluent (EFF); recycled activated sludge (RAS); and the river water column and sediments 100 m upstream (RU and SRU) and downstream (RD and SRD) of the WWTP discharge. Further details of the wastewater network, sampling regime, and sample handling and processing methods have been reported previously (Quintela-Baluja et al., 2019). Temperature, dissolved oxygen (DO), pH, and specific conductivity were measured in situ using hand-held probes (Mettler Toledo™, FG3 FiveGo™, and Jenway Model 350 pH Meter).

### 2.2 DNA extraction

Biomass was harvested for DNA extraction via vacuum filtration using sterile 0.22-μm membrane disc filters (Millipore, Billerica, MA, USA) or by centrifugation at 12,000 rpm for 30 min. Extraction was performed using the Fast DNA Spin Kit for Soils (MP Biomedicals, USA), according to the manufacturer's instructions. DNA was stored at −20°C prior to subsequent analysis.

### 2.3 Design of new primers and probes to quantify alternate clinical class 1 integron structures

The terminology of Gillings et al. (2008) has been used for many years to define class 1 integron recombination platforms. Pre-clinical class 1 integrons are elements that contain a class 1 integrase, which includes a complete tni transposition module (*pclintI1*). Conversely, "clinical" class 1 integrons (*clintI1*) contain *qacEΔ, sul1*, and *orf5* in their structure. A random subset of recently published plasmid sequences was examined, and it was determined the old primer set for *clintI1* poorly discriminated from *pclintI1* structures (Figure S1, Tables S1-S3). Therefore, we designed a new set of primers that are more specific to the *qacEΔ, sul1*, and *orf5* structure, which we call the *aint1* assay.

Our new name represents “anthropogenically impacted class 1 integrons” and is an appropriate name for class 1 integrons that have been impacted by human activity and not just clinical factors. In parallel we also designed a new assay for anthropogenic class 1 integrons that do not carry ARGs, which we call empty structures (*eaint1*). The design process of the new assays is provided under Results.

### 2.4 Quantitative PCR Assays and related data analysis

*IntI1, intI2*, and *intI3* as well as *aint1* and *eaint1* were quantified using the qPCR TaqMan method (Table 1). TaqMan qPCR reactions were conducted using SsoAdvanced™ Universal Probes Supermix (BioRad), employing the following thermocycler program: 3 min of initial denaturation at 95°C, and 40 cycles of 5 sec denaturation at 95°C and 30 sec annealing/extension (see temperatures in Table 1). In addition, total bacteria and total coliforms were quantified using a SYBR Green based qPCR assay. SYBR Green reactions were conducted using SsoAdvanced™ Universal SYBR® Green Supermix (BioRad), employing the following thermocycler program: 2 min of initial denaturation at 98°C, and 40 cycles of 5 sec denaturation at 98°C and 5 sec annealing/extension at 60°C (total bacteria) or 55°C (total coliforms).

**Table 1:** Primers used in this study to quantify integrons (*intI1, intI2, intI3*), anthropogenic class 1 integrons (*aint1*), *aint1* that contain no ARGs (*eaint1*), coliforms, and total bacteria (*16S rRNA*).

Target	Primer	Sequence (5′–3′)	Amplicon size (bp)	Annealing (°C)	Reference
*intI1*	F	GCCTTGATGTTACCCGAGAG	196	60	Barraud et al., 2010
	R	GATCGGTCGAATGCGTGT			
	Probe	(6FAM)ATTCCTGGCCGTGGTTCTGGGTTTT(BHQ1)			
*intI2*	F	TGCTTTTCCCACCCTTACC	195	60	Barraud et al., 2010
	R	GACGGCTACCCTCTGTTATCTC			
	Probe	(TxRd)TGGATACTCGCAACCAAGTTATTTTTACGCTG(BHQ2)			
*intI3*	F	GCCACCACTTGTTTGAGGA	138	60	Barraud et al., 2010
	R	GGATGTCTGTGCCTGCTTG			
	Probe	(6FAM)CGCCACTCATTCGCCACCCA(BHQ1)			
*eaint1*	F	GAGCAGCAACGATGTTAC	131	60	This study
	R	GGCTTATTATGCACGCTTA			
	Probe	(6FAM)CGCCCTAAAACAAAGTTAGATGCACT(BHQ1)			
*aint1*	F	GCACTAAGCACATAATTG	140	60	This study
	R	CCAACTATTGCGATAACA			
	Probe	(6FAM)GAGATATATCATGAAAGGCTGGCTT(BHQ1)			
Coliforms	Eco1457F	CATTGACGTTACCCGCAGAAGAAGC	190	60	Bartosch et al., 2004
	Eco1652R	CTCTACGAGACTCAAGCTTGC			
16S rRNA	1055f	ATGGCTGTCGTCAGCT	337	58	Harms et al., 2003
	1392r	ACGGGCGGTGTGTAC			

The limit of detection (LOD) and limit of quantification (LOQ) were calculated using a 2-fold dilution series covering the range of 1 to 500 gene copies (gc) from each standard and analysed eight times (R script available at https://github.com/cmerkes/qPCR_LOD_Calc). The LOD was defined as the lowest concentration that can be reliably detected, i.e., at which at least 95% of standard reactions are expected to amplify. The LOQ was defined as the lowest standard that can be quantified with a coefficient of variation less than 0.35 (Klymus et al., 2020) (Table S4).

All assays were carried out in triplicate using the BioRad CFX C1000 System (BioRad, Hercules, CA USA). To minimise inhibition, extracted DNA was diluted to a working concentration of 5 ng/ul and a DNA internal positive control was used in SYBR Green reactions. Briefly, an additional reaction was performed for each sample, containing 10^4^ genes copies of the green fluorescent protein gene (gfp). Inhibition was considered to be minimal when the difference in Ct values (ΔCt) of gfp between the spiked sample and the positive control was below 1 Ct. Standard curves for each primer set were constructed using plasmid clones of the target sequences, these were loaded in triplicate in each qPCR run, in parallel with the amplification of test samples. All samples were run in triplicate, using the optimum qPCR conditions determined for each primer set.

Related statistical analyses and data manipulation were performed using the R environment (Team, 2006) with a significant cutoff of α = 0.05. Normality and the variance homoscedasticity were tested by the Shapiro-Wilk test and Levene's test, respectively. Previous conditions were not met for all integron datasets. Therefore, the Krustall-Wallis test was performed to assess statistically significant differences. Conover's-test was performed for pairwise comparison between sampling sites, and adjusted p-values were calculated with the Holm-Bonferroni method.

### 2.5 16S rRNA gene amplification, sequencing, and data processing

To assess microbial communities, PCR amplification of the V4−V5 region of bacterial 16S rRNA genes in DNA extracts was conducted using fusion primers containing a PGM sequencing adaptor, a “GT” spacer, and a unique 12 base pair Golay barcode to allow multiplex analyses (primers 515F: 5′- GTGNCAGCMGCCGCGGTAA-3′, and 926R: 5′-CCGYCAATTYMTTTRAGTTT-3′). Details of PCR reactions and amplicon quantification are reported elsewhere (Quintela-Baluja et al., 2019).

Sequencing was performed using an Ion Torrent Personal Genome System (Life Technology) with processing performed using a UPARSE-QIIME pipeline (Pylro et al. 2014; Pylro et al., 2016). The Operational Taxonomic Unit (OTU) table was built using the UPARSE pipeline (Edgar, 2004). Sequences were clustered into OTUs at a 97% similarity cut off, checked for chimaeras, and reflective sequences were obtained for each phylotype. Taxonomic classification used QIIME (Caporaso et al., 2010) based on the uCLUST method (Prasad et al., 2015). See Quintela-Baluja et al. (2019) for details.

All data analysis and visualisations were conducted using R (Team 2006) through the Rstudio IDE (http://www.rstudio.com/). OTU counts and associated taxonomic assignments were imported and merged into a phyloseq object (McMurdie and Holmes, 2013). All samples were rarefied to ensure the same number of reads per sample (8,577), which corresponded to the sample with the fewest number of sequences, resulting in 5,698 unique OTUs.

Assessment of sample completeness (sample coverage) and alpha diversity were calculated with the R package iNEXT (Hsieh et al., 2016). Alpha-diversity was calculated using Hill numbers (D) with q from 0 to 3 and were expressed in units of “effective” number of species. As q increases, low-abundance OTUs are assigned less weight, while high-abundance OTUs are assigned more weight; this enables the investigation of diversity at different scales. At q=0, D is simply the number of observed OTUs (richness). At q=1, Dexp (Shannon), and at q=2, D is equal to inverse Simpson's index. A cluster dendrogram of community composition dissimilarity (Bray-Curtis, average neighbour clustering) was calculated with the R package vegan (Oksanen et al., 2019).

To determine the potential bacteria carrying mobile integrons (PBCMIs) in each compartment, consensus sequences from class 1 (anthropogenic and non-anthropogenic), 2, and 3 integrons were aligned against the NCBI_nr_ database (database accessed on June 2019) using BLAST® (McGinnis and Madden, 2004). Accession numbers from sequences with 100% identity were downloaded and taxonomy assigned with the R package taxonomizr. Only families with more than one entry in the NCBI_nr_ database were considered as potential integron hosts and used to filter the OTU table from the samples. The PBCMIs (OTU level) distribution across the network was visualized using a two-dimensional hierarchical clustering in conjunction with a heatmap of relative abundances.

### 2.6 Biomarker signature analysis

Linear discriminant analysis effect sizes (LEfSe) was employed to identify PBCMI that are statistically significant across sampling locations (Segata et al., 2011). With the normalised relative abundance matrix, LEfSe uses the Kruskal-Wallis rank-sum test to detect features with significantly different abundances between the assigned taxa and performs Linear Discriminant Analysis to estimate the effect size of each feature. Only taxa with average abundances >1% were considered significant. A significance level alpha of 0.05 and an effect size threshold of 2x were used for all the biomarkers evaluated in this study.

### 2.7 Co-occurrence between integrons and microbial taxa

A correlation matrix was developed by calculating all possible pairwise Spearman's rank correlations among 267 bacterial families, the three integrases (*intI1, intI2*, and *intI3*), and the 3’CS conserved region of *aint1* present in samples from the study (n = 27). A correlation between two items was considered if the Spearman's correlation coefficient (ρ) was ≥ 0.5 and the p value was ≤ 0.05. To reduce the chances of obtaining false-positive results, the p values were adjusted with a multiple testing correction using the Benjamini–Hochberg method (Benjamini and Hochberg, 1995). The pairwise correlations of the bacterial families and integrases formed their co-occurrence networks. Network analyses were performed in R environment and was further visualised and explored to identify its topological properties (i.e., clustering coefficient, shortest average path length and modularity) in Gephi (Bastian et al., 2009).

## 3 Results

### 3.1 New qPCR probe-primer sets for "human-impacted" and "empty" class 1 integrons

**Fig. 2:**
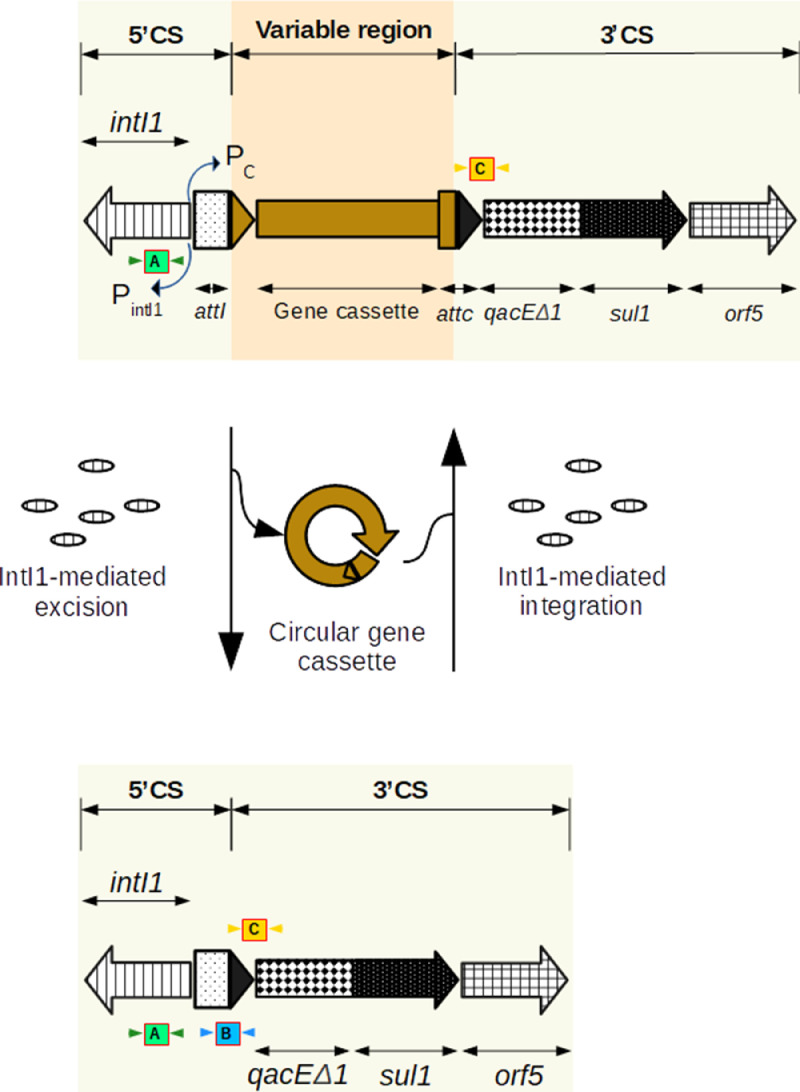
Anthropogenic class 1 integron structure with and without the variable gene cassette insertion region (top and bottom, respectively). The green, blue, and yellow boxes show the target sites of the respective primers:  - target for the class 1 integron (*clintI1*, Barraud et al., 2010):  - new target for the empty-class 1 integron structure (*eaint1*; this study); and  - new target location for the clinical class 1 integron at the attc/qacΔE1 interface (*aint1*; this study).

Although multiple probe-primers for class 1 integrons and associated gene cassettes already exist (Gillings et al., 2009; Gillings et al., 2009; Holmes et al., 2003; Stokes et al., 2001), one set has dominated use, i.e., clintI1 (hereafter called the “old set”) (Gillings et al., 2015). This old set targets the integron-integrase gene (5’CS end). However, when one compares that target sequence with more recent sequencing data, it becomes clear the region does not clearly discriminate between “clinical” and “pre-clinical” class 1 integrons (Figure S1) (pre-clinical here meaning the evolutionary precursor of clintI1). However, we identified a conserved 3’CS region (i.e., attc/qacΔE1/sul1) that is unique to clinical (i.e., human-impacted or anthropogenic) class 1 integrons (see Figs. 2 and S2, and Tables S1-S3 for details). Using this region, we designed and validated a new set of probe-primers that better segregate human-impacted class 1 from non-impacted class 1 structures (Table 1).

The assay design was as follows. Sequences of each individual region were first aligned using MUSCLE in UGENE v.33 to identify optimal target regions for more specific primers (Figure S2). This revealed selective targets within amplicon-containing sequences at intersecting loci between attc/*qac*Δ*E1* and *attI/attc* (Fig. 2). Using this region, primers and TaqMan probes were designed using OligoArchitect™ (http://www.oligoarchitect.com) with a melting temperature (Tm) of 60°C. The selected primers and probes were aligned against NCBI using BLAST® (McGinnis and Madden, 2004) to rule out non-specific binding to other bacterial targets. We call our new probe-primer assay *aint1* to avoid confusion with the old set. The new probe-primer set was assessed in terms of historic clinical and non-clinical integron structures (Tables S2 and S3), and shows *aint1* better targets the conserved 3’CS segment of the clinical structure (attc/qacΔE1/sul1) and does not overlap with pre-clinical structures. Henceforth we only refer to *aint1* hereafter.

Using a similar approach, a new probe-primer assay was also designed to quantify empty anthropogenic class I integron structures (which we call *eaint1*) (Table 1). The need to segregate between empty structures and integrons carrying GCs was evident as all of the empty class I integron structures in our plasmid sequence dataset were associated with anthropogenic class 1 structures. The old assay did not uniquely detect anthropogenic class 1 integron structures nor indicate whether they were carrying ARGs in the GC. Combining *aint1* and *eaint1* allows one to quantify anthropogenic class 1 integron dynamics more exactly, segregating GCs that do not contain ARGs.

### 3.2 Integron pool and distribution across our urban wastewater network

**Fig. 3:**
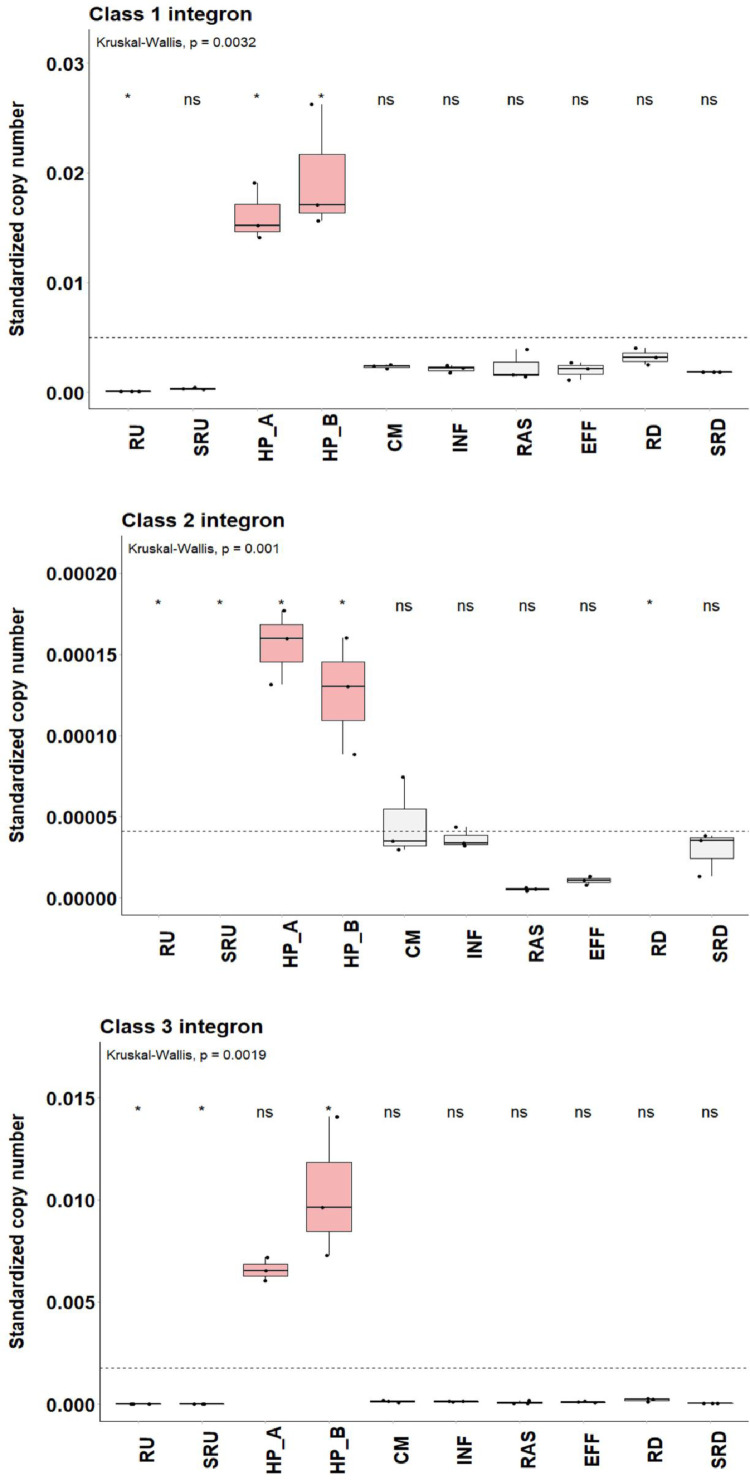
Comparison of the relative abundance of A) class 1; B) class 2; and C) class 3 integron genes per bacterial cell across the network. Krustall-Wallis rank sum test and the Wilcoxon-Mann-Whitney test was used to determine if each integron gene was enriched or reduced at each sample site compared with the population mean (dashed line). Significance: *0.05 and 'ns’ not significant. Sample sites: RU, upstream water column; SRU, upstream sediments; HP_A and HP_B, hospital wastewater; CM, community wastewater; INF, WWTP influent; RAS, recycled activated sludge; EFF, WWTP effluent; RD, downstream river water column; and SRD, downstream sediment.

Quantification of integron structures across our wastewater network showed that class 1 integrons were present in all compartments (Table 2 and S5; Fig. 3). In contrast, class 2 integron levels were low everywhere and below detection in river samples collected upstream of the wastewater effluent discharge point or in the downstream water column. Class 3 integrons were also low in upstream samples and lower across the wastewater network, except in hospital wastewater samples. Following quantification of integron gene levels per bacterial cell across the network (i.e., as genome equivalent), hospital effluents contained significantly higher levels of integron genes per cell (p < 0.05), much greater than any other compartment (Fig. 3, Table 2). Metadata on chemical conditions in each compartment is shown in Table S6.

**Table 2:** Estimated cell and coliform numbers, and relative total class 1 integron (*intI1*), anthropogenic class 1 integron (*aint1*), empty anthropogenic class 1 integron (*eaint1*), class 2 integron (*intI2*), and class 3 integron (*intI3*) per bacteria cell.

Sample	16S rRNA (copies/mL)^a^	Coliforms (copies/mL)	*intI1* (copies/cell)^b^	*aint1* (copies/cell)	*eaint1* (copies/cell)	*intI2* (copies/cell)	*intI3* (copies/cell)
RU	8.8 ± 0.11	0.24 ± 0.064	8.6E-5 ± 9.6E-6	1.6E-5 ± 1.2E-5	ND^c^	ND	1.4E-6 ± 2.4E-6
SRU	11.3 ± 0.052	0.12 ± 0.006	3.5E-4 ± 8.7E-5	2.9E-4 ± 6.8E-5	2.2E-5 ± 1.2E-6	ND	7.7E-6 ± 4.5E-6
HP_A	10.1 ± 0.053	3.2 ± 2.2	1.6E-2 ± 2.6E-3	7.9E-3 ± 1.3E-3	7.9E-4 ± 1.9E-4	1.6E-4 ± 2.3E-5	6.6E-3 ± 5.8E-4
HP_B	9.9 ± 0.038	5.7 ± 1.7	2.0E-2 ± 5.8E-3	1.3E-2 ± 3.4E-3	1.1E-3 ± 4.4E-4	1.3E-4 ± 3.6E-5	1.0E-2 ± 3.4E-3
CM	9.9 ± 0.14	2.3 ± 0.82	2.4E-3 ± 1.8E-4	1.2E-3 ± 1.1E-4	1.3E-4 ± 5.6E-5	4.6E-5 ± 2.4E-5	1.3E-4 ± 6.0E-5
INF	10.5 ± 0.37	0.59 ± 0.20	2.1E-3 ± 3.5E-4	1.2E-3 ± 2.7E-4	1.5E-4 ± 4.6E-5	3.6E-5 ± 6.3E-6	1.2E-4 ± 2.4E-5
RAS	11.7 ± 0.092	0.048 ± 0.013	2.3E-3 ± 1.4E-3	1.7E-3 ± 1.1E-3	5.4E-4 ± 4.0E-4	5.4E-6 ± 9.2E-7	7.8E-5 ± 6.8E-5
EFF	9.4 ± 0.031	1.3 ± 0.79	2.0E-3 ± 8.0E-4	1.4E-3 ± 6.0E-4	1.7E-4 ± 1.1E-4	1.1E-5 ± 2.7E-6	1.0E-4 ± 3.7E-5
RD	9.7 ± 0.056	0.38 ± 0.048	3.2E-3 ± 7.7E-4	1.9E-3 ± 6.5E-4	3.8E-4 ± 1.3E-4	ND	2.1E-4 ± 8.2E-5
SRD	11.6 ± 0.035	0.51 ± 0.17	1.9E-3 ± 6.3E-6	1.4E-3 ± 4.2E-5	3.1E-4 ± 1.4E-5	2.9E-5 ± 1.4E-5	2.7E-5 ± 7.9E-7

a On the basis of the Ribosomal RNA Database, the average number of 16S rRNA-encoding genes per genome equivalent was estimated as 4.1. 16S rRNA and coliform gene copy concentrations were divided by this value to estimate bacterial cell numbers or coliforms. For sediment samples, the denominator is sediment mass in gms rather than mL.

b The normalized copy numbers of integrons per bacterial cell were calculated by dividing the gene copy number of each integron by the estimated number of bacterial genome equivalents.

c ND refers to not detected genes in 5 ng/ul DNA.

Within the WWTP itself, integron abundances decreased between 93 to 98% from influent to effluent (log removals of 1.18 ± 0.28 [mean ± SD] for *intI1*, log 1.66 ± 0.26 for *intI2*, and log 1.21 ± 0.16 for *intI3*). These removals directly paralleled bacterial removals (Table 2 and S5), implying the removal of bacterial hosts and MGEs occur in tandem across the treatment steps. This is confirmed by *intI1, aint1*, and *intI3* copies per bacterial cell being roughly constant across the system (Table S7). Despite large reductions in integrons across the WWTP, absolute class 1 and 3 integron gene abundances increased in the river downstream of the WWTP, suggesting integrons were still reaching the environment.

**Fig. 4:**
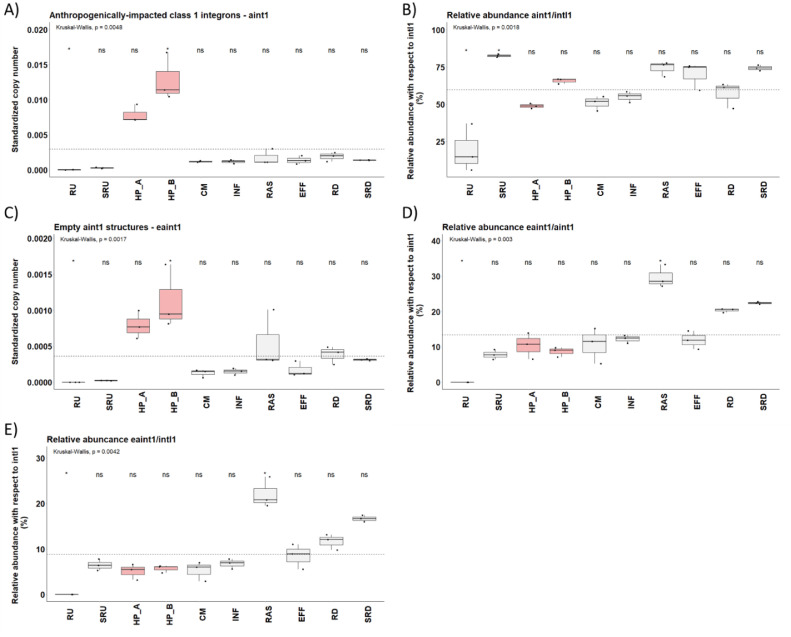
Relative abundances of class 1 integron genes of different types. A) Relative number of *aint1* per bacteria cell; B) Relative abundance of *aint1* structure per *intl1* gene; C) Relative number of *eaint1* per bacteria cell; D) Relative abundance of *eaint1* per *aint1*; and E) Relative abundance of *eaint1* per *intl1*. Krustall-Wallis rank sum test and the Wilcoxon-Mann-Whitney test was used to determine if each integron structure was enriched or reduced at each sample site compared with the population mean (dashed line). Significance: *0.05 and 'ns’ not significant. Sample sites: RU, upstream water column; SRU, upstream sediments; HP_A and HP_B, hospital wastewater; CM, community wastewater; INF, WWTP influent; RAS, recycled activated sludge; EFF, WWTP effluent; RD, downstream river water column; and SRD, downstream sediment.

The new probe-primer design showed that the ratio of *aint1* to *intI1* did vary across "raw" wastewater compartments (Fig. 4A). *aint1* was the dominant integron structure downstream of the biological wastewater treatment unit (p <0.05) (Fig. 4B), with relatively low levels of *eaint1*. In contrast, the percentage of *eaint1* in RAS was consistently higher, double that in WWTP influents and hospital samples, and much higher than any other compartment (Fig. 4C-D), except downstream river locations.

### 3.3 Identifying potential bacteria carrying mobile integrons (PBCMI)

Integrase gene abundance across this wastewater network correlates with the type and abundance of bacterial populations, which was previously observed for ARGs within this network (Quintela-Baluja et al., 2019). Therefore, data here lends itself well to statistical analysis on most probable populations carrying key integrons, especially *intI1* vs *aint1* vs *eaint1*.

Screening of the NCBI_nr_ database found 28 potential bacterial families known to host *intI1*: 14 for *aint1*, 6 for *intI2*, and 4 for *intI3* (Table S8)*.* Only *intI1* has been previously found in Gram-positive bacteria, whereas the 3’CS conserved region of *aint1* has only been found in Gram-negative bacteria. Of the identified families with mobile integrons (from the database), 21 were found in one or more samples in this study (Table S8).

Beta-diversity analysis was used to compare microbial community diversities among wastewater network locations. For this analysis, the dataset was re-sampled to ensure the same number of reads per sample (8,577), which corresponded to the sample with the fewest number of sequences, resulting in 5,698 OTUs. The OTUs from PBCMI families were filtered and re-sampled (from the raw reads) to the sample with the fewest number of sequences (1,266), resulting in 392 unique OTUs. The percentage of total estimated species richness in each sample resulted in 95.4 - 99.0% for the total microbial community, and 94.4 - 98.1% for the PBCMI, which suggests the overall microbial communities were adequately sampled in both cases (Table S9).

Hill number diversity indices were calculated with the parameter q from 0 to 2. Note that as q increases, rare species are given less weight and contribute less toward the “effective number of OTUs” in a sample. Hill numbers with q equal to 0 and 1 showed that raw wastewater samples had lower diversity compared with upstream river samples (both water column and sediment), and from the downstream river sediments (Fig. S3) Specifically, Hill numbers (q equal to 0 and 1) indicated that bacterial diversity was greater in samples upstream of the WWTP (RU, SRU) because of more rare taxa, which was not apparent when only PBCMI taxa were considered (Fig. S4).

Conversely, the Bray-Curtis dissimilarity dendrogram showed that total microbial community compositions differed among network compartments, which included four main clusters with distinct ecosystems (cut off = 0.72) (Fig. S5). The first cluster contained samples associated with raw wastewater (community, hospital, and WWTP influent). The second cluster contained RAS, WWTP effluent, and the downstream river water and sediment samples. The third and fourth clusters included the river water column and sediment samples upstream of the WWTP, respectively.

**Fig. 5:**
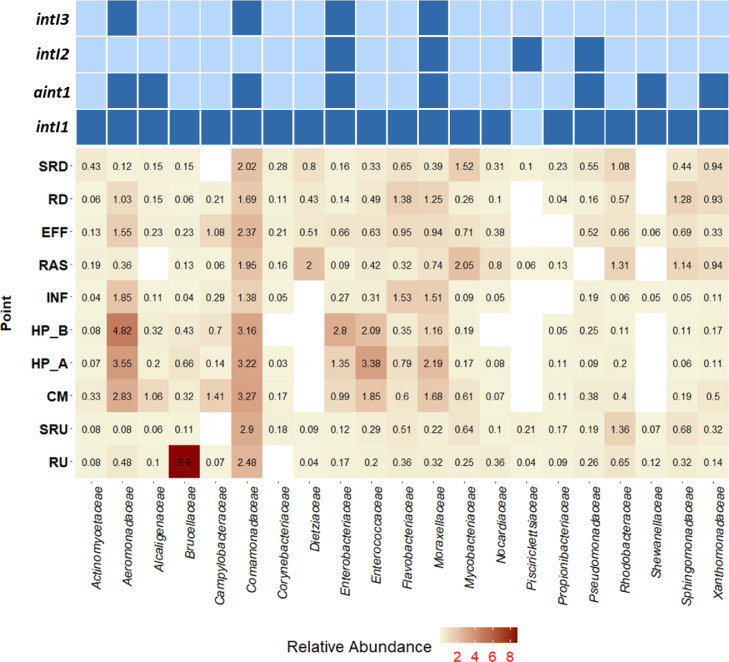
Relative abundance of potential bacteria carrying integron genes in wastewater network compartments. The right side of the table shows the bacterial families which host integron genes (dark blue). The numbers show the relative abundance of the potential bacteria hosting integrons in each compartment. RU, upstream river water column; SRU, upstream river sediments; HP, hospital wastewater (HP_A and HP_B); CM, community wastewater; INF, WWTP influent; RAS, recycled activated sludge; EFF, WWTP effluent; RD, downstream river water column; and SRD, downstream river sediment.

The PBCMI sub-community followed similar patterns, but the cut off value of the cluster structure was significantly lower (cut off = 0.53). This observation, in conjunction with evident two-dimensional hierarchical clustering seen in the heatmap of PBCMI relative abundances (Fig. S6), suggests that a PBCMI core sub-community exists in all network compartments. However, Fig. S6 shows that WWTP releases clearly impact river microbial communities, increasing PBCMIs in the downstream environment (Figs. 5 and S6). The family *Comamonadaceae* is ubiquitous, which is often associated with wastewater (e.g., *Aeromonadaceae* and *Enterobacteriaceae*), and those more associated with the environment (e.g., *Rodobacteriaceae* or *Sphingomonadaceae*).

### 3.4 Microbial communities and their connectivity across the network

Characterising microbial communities across a wastewater network (in terms of diversity, evenness, and taxonomic composition) is key to identifying linkages among compartments and determining microbial contributions from internal versus external sources. LEfSE analysis showed the relative presence of the family *Erysipelotrichaceae, Lachnospiraceae, Bacteroidaceae,* and *Ruminococcaceae* tend to best define community wastewater. In contrast, hospital wastewater was characterised by higher abundances of *Prevotellaceae, Enterobacteriaceae, and Desulfovibrionaceae. Weeksellaceae* and *Moraxellaceae* were associated with WWTP influent, whereas RAS was best defined by *Rhodobacteraceae, Frankiaceae, Dietziaceae, Gordoniaceae*, and *Intrasporangiaceae* (Fig. S7).

Each compartment of the network had characteristic, reflective signature families, suggesting local enrichment and-or selection. To examine this, we combined the biomarker discovery tool LefSE and co-occurrence network analysis to assess the relative transmission of ARGs associated with mobile integrons across compartments. The co-correlation network consists of 259 nodes (255 taxa and 4 mobile integrons) and 4.465 edges with an average degree or node connectivity of 34.479. The average network distance between all pairs of nodes (average path length) was 2.94 edges with a network diameter of 7 edges.

Bacterial families, comprising PBCMI, that most explain differences between network compartments (LefSE analysis) are highlighted in Fig. S7. Network analysis produced four different modules (Fig. S8), equivalent to the microbial composition dissimilarity clusters. The network showed possible ARG dissemination pathways via mobile integrons from human-associated taxa (Module I) to bacteria in human-impacted environments (Module II) and non-human environmental bacteria (Modules III and IV). LEfSE analysis revealed that Module I include bacterial families defining raw wastewater (CM, HP, and INF) and all mobile integrons (Fig. S9A). Conversely, Module II mostly contains RAS and river sediment taxa with limited PBCMI contributions (RAS, RD, SRD, and EFF), emphasising the connectivity of RAS bacteria with river sediments (Fig. S8B). Module III contains taxa defining the river water column (Fig. S8C). Finally, Module IV contains mostly bacterial families in upstream river sediments (SRU). However, some bacteria families from the downstream river sediments also are present in Module IV (Fig. S8D).

## 4 Discussion

Here we show that *intI1* and *aint1* dominated all compartments in our wastewater network, with *intI2* and *intI3* being much less abundant at all sampling points. All integron markers, except for empty structures (*eaint1*), were highest in hospital wastewater where antibiotic use is more intensive (Escudero-Oñate et al., 2017; Rodriguez-Mozaz et al., 2015). This is consistent with previous data that showed the relative number of ARGs per bacterial cell were at least one order of magnitude higher in hospital wastewater compared with the other compartments (Quintela-Baluja et al., 2019). Interestingly, while *intI2* levels per bacterial cell declined along the wastewater process stream, the relative levels of *intI1, aint1*, and *intI3* per cell did not change amongst compartments, except in upstream river samples. Consistently low levels of *intI2* are probably due to it typically lacking a functional integrase, meaning it cannot catalyse recombination reactions alone (Deng et al., 2015).

It is noteworthy that *intI3* was detected across the whole wastewater network and roughly mirrored *intI1* patterns, although abundances were about 20 times lower than *intI1.* The ubiquitous presence of *intI3* here contrasts with results of Stalder et al. (2014) who found *intI3* levels decreased across their WWTP. This may be due to differences in *intI3* found in the two studies or differences between the local wastewater systems. *IntI3* performs a similar function to *intI1*, although its recombination frequencies have historically been lower (Stokes and Gillings, 2011), which makes *intI3* less associated with bacterial adaptation under adverse conditions.

Empty human-impacted class 1 integron structures have been found previously (Malek et al., 2015; Mokracka et al., 2012; Moura et al., 2012; Sá et al., 2010; Toval et al., 2015), but here we more exactly quantified levels of *eaint1* vs *aint1* and *intI1*. We had expected to find higher relative abundances of *eaint1* cassettes in hospital wastewater. It was hypothesized that the presence of higher concentrations of antibiotics and other bioactive agents would select for cellular defence GCs and trigger SOS responses that would activate integrase *aint1* expression (Cambray 2011). But instead, the highest relative abundances of *eaint1* cassettes were found in RAS samples, suggesting this is where empty cassettes accumulate or, more likely, ARG carriage is not a selection factor for bacterial hosts that reside in RAS. RAS appears to have a reduced tendency for class 1 integrons or their hosts to retain ARGs.

Although the scale of differences may be specific to our network, we suggest hospital wastewaters tend to select and retain a greater array of “defence” genes in their *aint1* due to greater stressors in their environment (e.g., therapeutic drugs) and-or richer organic carbon levels (wastes dominated by faeces from patients). Similar uniqueness of hospital wastewater was also seen in Sweden (Kraupner et al., 2021). Conversely, bacteria in RAS grow under nutrient-limited conditions and under longer residence times (e.g., than a gut environment); thus, RAS bacteria are under persistent nutrient stress compared to the gut. Most activated sludge WWTPs recycle RAS for days rather than hours under comparatively dilute but fluctuating biotic and abiotic conditions. Such conditions might promote rearrangement in *aint1* and loss of ARGs, but apparently not reacquisition, possibly because selective agents (e.g., antibiotics) are more dilute or not bioavailable.

The high prevalence of empty cassettes in RAS and their apparent low presence in the WWTP effluents has important implications to pathways of integron-mediated ARG spread from WWTPs to the environment. Until now, only two studies have used high-throughput sequencing to study *intI1* cassette diversity in environmental samples (Gatica et al., 2016; Stalder et al., 2014). While Stalder et al. (2014) sequenced clone libraries of *intI1* cassettes from wastewater compartments, Gatica et al. (2016) targeted recombination sites that flank the gene-cassettes of *intI1* genes in WWTP effluents. Both studies found a high diversity of gene cassettes; however, many of their detected sequences had unknown functions. Stalder et al. (2014) found their WWTP tended to reduce the diversity of gene cassette arrays relative to raw wastewater and had elevated levels of empty cassettes. Whereas Gatica et al. (2016) showed a marginal persistence of β-lactam resistance gene cassettes in soil microbiomes impacted by WWTP effluents.

Work here shows the nature and content of integron structures, and associated microbial communities differ widely across compartments, further confirming “local” evolutionary ecosystems drive selection in sub-ecosystems embedded within larger networks (Baquero et al., 2008; Quintela-Baluja et al., 2019). Local ecosystems and their communities are spatially separate, and each is driven by different habitat and other selective factors. This includes specific community selection (Munck et al., 2015) and ARG carriage (Quintela-Baluja et al., 2019). Parallel differences exist among prevalent mobile integrons, and their state relative to ARG carriage (empty or not). However, even though different wastewater compartments have different communities, continual hydraulic interconnection allows microbial dispersal and gene flow, which explains linkages among local resistomes, including local mobile integrons.

Co-occurrence analysis (Fig.s S8 and S9) highlights the association of PBCMIs within microbial communities in raw wastewater, especially compared with communities less impacted by raw wastewater*.* The importance of this observation is most evident in river sediments, where resident bacteria and their mobile integrons reflect unsettled floc in WWTP effluents. As such, sediments may be place of environmental ARG transfer, although there would need to be selective drivers for gene acquisition to occur, such as other pollutants in the sediments. Our previous work supported this hypothesis where downstream sediments were largely comprised of RAS bacteria (Quintela-Baluja et al., 2019). A recent study by Di Cesare et al. (2020) supported this hypothesis by showing that the vertical distribution of *intI1* in lake sediments mirrored the deposition of bacteria from the water column and to sediments below.

Consistent with previous work, sediments downstream of our WWTP were enriched in ARGs, probably due to outputs from the WWTP (Di Cesare et al., 2020; Quintela-Baluja et al., 2019), which is consistent with greater abundances of *aint1* compared with upstream sediments. Although it is speculation, we suspect the co-release of potentially selective agents in WWTP effluents might further promote such persistence. Specifically, the dynamic process of gene cassette acquisition in *aint1* and the presence of a *qac* gene in its structure (resistance to biocides) potentially confers advantageous phenotypes that may be behind the fixation and spread of this integron structure (Gillings et al., 2009).

A noteworthy new observation here, which supports the potential for ARG transfer in sediments downstream of WWTPs, is elevated levels of *eaint1.* The two compartments with the highest relative levels of empty clinical class 1 integron cassettes are the RAS and downstream sediments, which implies they are hydraulically connected and may be locations of reacquisition of ARGs due to higher levels of empty mobile integrons. A comparison of microbial communities between RAS and downstream sediments (Quintela-Baluja et al., 2019) furthers shows this connection and suggests downstream sediments could be locations of elevated horizontal gene transfer.

## 5 Conclusions

Here we show that integrons have their own ecology within a wastewater network and can be used to speculate on the dynamics of ARGs within such systems. However, integron dynamics appear more complex than previously reported, especially the spatial distribution of ARGs, and relationships with human-impacted and empty class 1 integron cassettes. This recognition was only possible by developing a more exact probe-primers for anthropogenic class 1 integrons that better segregate class 1 integron types and determine whether they carry ARGs or not. We are clearly getting closer to understanding what really happens in wastewater networks relative to AMR spread, but new evidence here suggests ARG transmission in wastewater networks may differ than previously beleived. Regardless, we hope this work will guide new and improved WWTPs and their operations in the future.

## Data availability

The 16S rRNA sequencing data were submitted to EBI with accession number PRJEB46784 http://www.ebi.ac.uk/ena/data/view/PRJEB3216.

## Declaration of Competing Interest

NA.

## Appendix Supplementary materials

Image, application 1
