# Supplementary material for: Dynamics of integron structures across a wastewater network – Implications to resistance gene transfer
Source: Water Res. 2021 Nov 1;206:117720. doi: 10.1016/j.watres.2021.117720 (PMC8626773; doi:10.1016/j.watres.2021.117720)
Supplement: Supplementary file 1 [file mmc1.docx]

# Supplementary Data

Dynamics of integron structures across a wastewater network – Implications to resistance gene transfer

Marcos Quintela-Baluja^1,2#^, Dominic Frigon^3^, M. Abouelnaga^4^, Kelly Jobling^1^, Jesús L. Romalde^5^, Mariano Lopez Gomez^6^, David W. Graham^1#^

^1^School of Engineering, Newcastle University, Newcastle upon Tyne, UK

^2^Department of Analytical Chemistry, Nutrition and Bromatology, University of Santiago de Compostela, Spain

^3^Department of Civil Engineering and Applied Mechanics, McGill University, Montréal (QC), Canada

^4^Department of Analytical Chemistry, School of Veterinary Sciences, Suez Canel University, Ismailia, Egypt

^5^Departamento de Microbiología y Parasitología, CIBUS-Facultad de Biología & Institute CRETUS, Universidade de Santiago de Compostela, Santiago de Compostela, Spain

^6^Labaqua, Santiago de Compostela, Spain

^#^Contributed equally: Technical correspondence to Dr Marcos Quintela-Baluja ([marcosquintelab@gmail.com](mailto:marcosquintelab@gmail.com)) and general correspondence to Prof David W Graham.

Submission by: Prof David W. Graham

School of Engineering

Cassie Building

Newcastle University

Newcastle upon Tyne

United Kingdom NE1 7RU

Phone: +44-191-208-7930

E-mail: [david.graham@newcastle.ac.uk](mailto:david.graham@newcastle.ac.uk)

**Summary of Supporting Tables and Figures**

**Tables:**

**Table S1:** Consensus sequences used to identify potential bacteria carrying class 1, class 2, class 3, and class 1 clinical integron from the NCBI database.

**Table S2:** Set of primers used in current and previous class 1 integron studies. The primers were evaluated using a set of plasmid sequences downloaded from NCBI.

**Table S3:** Sequence matches with a random subset of plasmid sequences and previously published sets of primers (see Table S2).

**Table S4:** qPCR control data and limit of detection and limit of quantification of the assays.

**Table S5:** Gene concentrations at each sampling site (log (copies per ml or gram)) for 16S rRNA, Coliforms, integrons (intI1, intI2, intI3), anthropogenic class 1 integrons (*aint1*), and empty anthropogenic class 1 integron structures (eaint1)

**Table S6:** Metadata from different sampling sites: RU) Water column river upstream STP, SRU) Sediment river upstream STP, HP_A and HP_B) Hospital sewage, CM) Community sewage, INF) Influent STP, RAS) Returning activated sludge, EFF) Effluent STP, RD) water column river downstream, SRD) sediment river downstream. Measurements from three different weeks for pH, conductivity, D.O, temperature, COD, DNA concentration and bacteria density.

**Table S7:** A) Data and statistical analysis of normalized data per bacterial cell for class 1 integron. B) Data and statistical analysis of normalized data per bacterial cell for class 2 integron. C) Data and statistical analysis of normalized data per bacterial cell for class 3 integron. D) Data and statistical analysis of normalized data per bacterial cell for anthropogenic class 1 integron structures (aint1). E) Data and statistical analysis of normalized data per bacterial cell for empty class 1 anthropogenic class 1 integrons (eaint1). F) Data and statistical analysis of relative clinical class 1 integron per intI1 gene. G) Data and statistical analysis of relative empty clinical class 1 integron per clinical class 1 integron.

**Table S8:** Diversity of bacteria families carrying integron classes 1, 2, 3 or clinical class 1 integrons.

**Table S9:** Sample coverage. The percentage of total estimated species richness in a sample for the sample’s microbial community and the PBCMI.

**Figures:**

**Figure S1:** Multiple sequence alignment tree of the integrase sequence from integrases from anthropogenically impacted class 1 integron structures (clintI1) and integrases from non clintI1 (pclintI1) structures. We define pre-clinical class 1 structures is the evolutionary pre-cursor to clintI1. Clustering shows that the integrase sequence does not discriminate between clintI1 and pclintI1 structures.

**Figure S2:** A) Conserved region from the clinical class 1 integron (i.e., qacEΔ1/sul1 region). Sequences were aligned in Clustal X 2.0, and primers and TaqMan probe were designed in this region (aint1). B) Conserved region from the empty clinical class 1 integron. Sequences were aligned in Clustal X 2.0, and primers and TaqMan probe were designed in this region (eaint1).

**Figure S3:** Diversity profile curves of microbial communities, using plots of Hill numbers q D(') as a function of order q, 0 q 3.

**Figure S4:** Diversity profile curves of PBCMI, which plots Hill numbers q D(') as a function of order q, 0 q 3.

**Figure S5:** Cluster dendrograms of community composition dissimilarity (Bray-Curtis, average neighbour clustering) based on A) OTU distributions (all OTUs), and B) OTUs only from PBCMI families among sampling sites.

**Figure S6:** Heatmap and cluster analysis (Bray–Curtis; complete linkage) of PBCMI (OUT level) for different sampling points. Relative abundances are log-transformed for visualization purposes.

**Figure S7:** A linear discriminant analysis effect size (LEfSe) method identifies the significantly different abundant taxa of bacteria in all the sampling sites (family level). Each bacteria family has an effect size LDA score. Only taxa meeting an LDA significance threshold of >2 are shown. Samples are grouped by sampling site.

**Figure S8:** A network analysis revealing the co-occurrence bacteria taxa (family level) and MIs. Connections represent a Spearman’s correlation coefficient p > 0.4 and significant correlation (p-value > 0.05). The PBCMI are highlighted in red text. The network shows the presence of four different modules, which are highlighted in different colours. Module I primarily represent bacteria from raw wastewater sources (CM, HP, INF), module II represents bacteria from the WWTP or the downstream water column (RAS, EFF, RD, SRD). Taxa in Module III are from the upstream water column (RU) and Module IV river sediments (SRU)

**Figure S9:** A) Module I represent mostly bacteria from the wastewater sources (i.e., CM, HP, INF) and contain all the detected MIs. B) Module II primarily represents bacteria from human-impacted environments (RAS, SRD). C) Module IV represents mostly bacteria from the water column from the river upstream (RU). D) Module III represents mostly bacteria from non-human environmental bacteria (SRU).

**Table S1:** Consensus sequences used to identify potential bacteria carrying class 1, class 2, class 3, and class 1 clinical integron from the NCBI database.

**Table S2:** Set of primers used in current and previous class 1 integron studies. Also included are our new anthropogenic class 1 integron structures, The primers were evaluated using a set of plasmid sequences downloaded from NCBI.^a^

| Primer Name | Sequence (3'-5') | Target region | Source |
| --- | --- | --- | --- |
| hep58 | TCATGGCTTGTTATGACTGT | clintI1 cassette array | White et al. 2001 |
| hep59 | GTAGGGCTTATTATGCACGC |  |  |
| HS549 | ACTAAGCTTGCCCCTTCCGC | sul1 gene | Marquez et al. 2008 |
| HS550 | CTAGGCATGATCTAACCCTCGG |  |  |
| HS458 | GTTTGATGTTATGGAGCAGCAACG | 5'-CS attI1 end 3'-CS | Holmes et al. 2003 |
| HS459 | GCAAAAAGGCAGCAATTATGAGCC |  |  |
| HS463 | CTGGATTTCGATCACGGCACG | *intI1* gene | Holmes et al. 2003 |
| HS464 | ACATGCGTGTAAATCATCGTCG |  |  |
| 3CS | AAGCAGACTTGACCTGA | clintI1 cassette array | Levesque et al. 1995 |
| 5CS | GGCATCCAAGCAGCAAG |  |  |
| MRG284 | GTTACGCCGTGGGTCGATG | non-clinical *intI1* cassette array | Gillings et al. 2009 |
| MRG285 | CCAGAGCAGCCGTAGAGC |  |  |
| aint1F | GAGCAGCAACGATGTTAC | 3'-CS | This study |
| aint1R | CCAACTATTGCGATAACA |  |  |
| intI1F165 | CGAACGAGTGGCGGAGGGTG | *intI1* gene | Gillings et al. 2015 |
| IntI1R476 | TAC CCGAGAGCTTGGCACCCA |  |  |
| intI1LC1 | GCCTTGATGTTACCCGAGAG | *intI1* gene | Barraud et al. 2010 |
| intI1LC5 | GATCGGTCGAATGCGTGT |  |  |

*^a^* The primers HS464/HS463a targeting a conserved region of 473 bp of the class 1 integrase where to download class 1 recombination platforms from the non-redundant GenBank sequence database of the National Center for Biotechnology Information (NCBI) (<http://www.ncbi.nlm.nih.gov/>). The primers MRG284/MRG285 were used to identify pre-clinical gene cassettes amplifying the region between the class 1 integrase and immediately after the most distal attC site. Primers HS458 and HS459 were used to identify clinical gene cassettes amplifying the region between the class 1 integrase and the 3’ conserved segment (3’-CS). The integrase sequences from the screened clinical and pre-clinical class 1 integron recombination platforms were aligned with MUSCLE (Edgar, 2004), and a multiple sequence alignment tree was conducted in UGENE v.33, using the neighbour joining method with 1000 bootstrap replicates to construct distance-based trees. The phylogenetic tree visualisation was performed with the R package ggtree (Yu et al. 2017).

**Table S3**: Sequence matches with a random subset of plasmid sequences and previously published sets of primers (see Table S2).

| Genus | Accession number | hep58-hep59 | HS549-HS550 | HS458-HS459 | HS463-HS464 | 3CS-5CS | MRG284-MRG285 | aint1F-aint1R | intI1F165-IntI1R476 | intI1LC1-intI1LC5 | qacEΔ1-sul1 |
| --- | --- | --- | --- | --- | --- | --- | --- | --- | --- | --- | --- |
| *Pseudomonas* | MK388092 | NO | NO | NO | YES | NO | YES | NO | YES | YES | NO |
| *Enterobacter* | MF344583 | NO | NO | NO | YES | NO | YES | NO | YES | YES | NO |
| *Enterobacter* | MF344574 | NO | NO | NO | YES | NO | YES | NO | YES | YES | NO |
| *Klebsiella* | CP018887 | NO | NO | NO | YES | NO | YES | NO | YES | YES | NO |
| *Klebsiella* | CP018884 | NO | NO | NO | YES | NO | YES | NO | YES | YES | NO |
| *Pseudomonas* | LT969519 | NO | NO | NO | YES | NO | YES | NO | NO | YES | NO |
| *Klebsiella* | CP021699 | NO | NO | NO | YES | NO | YES | NO | YES | YES | NO |
| *Citrobacter* | KX784503 | NO | NO | NO | YES | NO | YES | NO | YES | YES | NO |
| *Klebsiella* | KX784502 | NO | NO | NO | YES | NO | YES | NO | YES | YES | NO |
| *Pseudomonas* | LC075716 | NO | NO | NO | YES | NO | YES | NO | YES | YES | NO |
| *Klebsiella* | KT070138 | NO | NO | NO | YES | NO | YES | NO | YES | YES | NO |
| *Klebsiella* | KT345947 | NO | NO | NO | YES | YES | YES | NO | YES | YES | NO |
| *Pseudomonas* | KR106190 | NO | NO | NO | YES | NO | YES | NO | YES | YES | NO |
| *Pseudomonas* | KP873172 | NO | NO | NO | YES | NO | YES | NO | YES | YES | NO |
| *Pseudomonas* | LC054840 | NO | NO | NO | YES | NO | YES | NO | YES | YES | NO |
| *E. coli* | CP022156 | NO | NO | NO | YES | NO | YES | NO | YES | YES | NO |
| *Enterobacter* | KY126370 | NO | NO | NO | YES | NO | YES | NO | YES | YES | NO |
| *Pseudomonas* | KJ463833 | NO | NO | NO | YES | NO | YES | NO | YES | YES | NO |
| *Serratia* | KJ577613 | NO | NO | NO | YES | NO | YES | NO | YES | YES | NO |
| *Pseudomonas* | KF840720 | NO | NO | NO | YES | NO | YES | NO | YES | YES | NO |
| *Pseudomonas* | KF040452 | NO | NO | NO | YES | NO | YES | NO | YES | YES | NO |
| *Pseudomonas* | KC821786 | NO | NO | NO | YES | NO | YES | NO | YES | YES | NO |
| *Salmonella* | JN983043 | NO | NO | NO | YES | NO | YES | NO | YES | YES | NO |
| *Klebsiella* | MG764534 | YES | YES | YES | YES | YES | NO | YES | YES | YES | YES |
| *Pseudomonas* | CP040126 | YES | YES | YES | YES | YES | NO | YES | YES | YES | YES |
| *Providencia* | CP031122 | YES | NO | YES | YES | YES | YES | YES | YES | YES | YES |
| *Pseudomonas* | KC543497 | YES | YES | YES | YES | YES | NO | YES | YES | YES | YES |
| *E. coli* | CP040263 | YES | YES | YES | YES | YES | NO | YES | YES | YES | YES |
| *Klebsiella* | CP043970 | YES | YES | YES | YES | YES | NO | YES | YES | YES | YES |
| *E. coli* | CP043943 | YES | NO | YES | YES | YES | NO | YES | YES | YES | YES |
| *E. coli* | CP043947 | YES | NO | YES | YES | YES | NO | YES | YES | YES | YES |
| *Klebsiella* | CP043927 | YES | YES | YES | YES | YES | NO | YES | YES | YES | YES |
| *E. coli* | CP043741 | YES | YES | YES | YES | YES | NO | YES | YES | YES | YES |
| *Serratia* | MK123268 | YES | YES | YES | YES | YES | NO | YES | YES | YES | YES |
| *E. coli* | MK123267 | YES | YES | YES | YES | YES | NO | YES | YES | YES | YES |
| *Salmonella* | MN241904 | YES | YES | YES | YES | YES | NO | YES | YES | YES | YES |
| *E. coli* | CP022227 | YES | YES | YES | YES | YES | NO | YES | YES | YES | YES |
| *E. coli* | KU043115 | YES | YES | YES | YES | YES | NO | YES | YES | YES | YES |
| *Klebsiella* | CP018669 | YES | YES | YES | YES | YES | NO | YES | YES | YES | YES |
| *E. coli* | CP010232 | YES | NO | YES | YES | YES | NO | YES | YES | YES | YES |
| *E. coli* | CP010174 | YES | YES | YES | YES | YES | NO | YES | YES | YES | YES |
| *Aeromonas* | CP018201 | YES | YES | YES | YES | YES | NO | YES | YES | YES | YES |
| *Salmonella* | CP016013 | YES | YES | YES | YES | YES | NO | YES | YES | YES | YES |
| *Salmonella* | LN829403 | YES | NO | YES | YES | YES | NO | YES | YES | YES | YES |
| *Salmonella* | CP009414 | YES | NO | YES | YES | YES | NO | YES | YES | YES | YES |
| *Bordetella* | KF743818 | YES | YES | YES | YES | YES | NO | YES | YES | YES | YES |

**Table S4:** qPCR control data and limit of detection and limit of quantification of the assays

| Assay | R.squared | Slope | Intercept | LOD | LOQ |
| --- | --- | --- | --- | --- | --- |
| *aint1* | 0.988 | -3.72 | 40.7 | 11.3 | 96.0 |
| *aeint1* | 0.986 | -3.29 | 38.8 | 25.2 | 25.2 |
| *intI1* | 0.975 | -3.25 | 40.8 | 16.3 | 128.0 |
| *intI2* | 0.992 | -3.56 | 41.7 | 12.9 | 90.0 |
| *intI3* | 0.986 | -3.25 | 40.2 | 12.9 | 47.0 |
| 16S rRNA | 0.996 | -3.19 | 40.5 | 18.4 | 51.0 |
| coliforms | 0.984 | -3.41 | 42.0 | 17.5 | 53.0 |

**Table S5:** Gene concentrations at each sampling site (log (copies per ml or gram)) for 16S rRNA, Coliforms, integron 1, 2, and 3, anthropogenic class 1 integron, and empty anthropogenic class 1 integron.

Note: RU= upstream river water column; SRU = upstream river sediment; HP_A, HP_B = wastewater from two different hospitals; CM = community wastewater; INF = wastewater treatment plant (WWTP) influent; RAS = recycled activated sludge; EFF = WWTP effluent; RD = downstream river water column; and SRD = downstream river sediment.

**Table S6:** Metadata from different sampling sites: RU) Water column river upstream STP, SRU) Sediment river upstream STP, HP_A and HP_B) Hospital sewage, CM) Community sewage, INF) Influent STP, RAS) Returning activated sludge, EFF) Effluent STP, RD) water column river downstream, SRD) sediment river downstream. Measurements from three different weeks for pH, conductivity, D.O, temperature, COD, DNA concentration and bacteria density.

| Samples | pH | Conductivity (μS/cm) | DO (% of air saturation) | Temperature (^o^C) | COD (mg/L or mg/gm) | DNA concentration (ng/mL) | Bacteria density log (cells /mL) |
| --- | --- | --- | --- | --- | --- | --- | --- |
| RU | 7.6 | 101 | 86 | 14.5 | 8 | 19 | 8.32 |
|  | 7.27 | 105 | 88 | 15.7 | 6 | 21 | 8.11 |
|  | 7.49 | 125 | 47.7 | 15 | 9 | 24 | 8.22 |
| SRU | 7.6 | 101 | 86 | 14.5 | 410 | 15310 | 10.6 |
|  | 7.27 | 105 | 88 | 15.7 | 580 | 19790 | 10.7 |
|  | 7.49 | 125 | 47.7 | 15 | 700 | 18750 | 10.7 |
| HP_A | 7.97 | 513 | 54 | 24 | 1070 | 93 | 9.54 |
|  | 8.78 | 560 | 51.7 | 22.6 | 1600 | 97 | 9.50 |
|  | 8.22 | 422 | 42.5 | 23 | 1010 | 93 | 9.43 |
| HP_B | 7.7 | 398 | 57 | 23.7 | 829 | 70 | 9.30 |
|  | 7.75 | 587 | 38 | 22.5 | 890 | 85 | 9.22 |
|  | 7.6 | 624 | 54 | 21.9 | 843 | 78 | 9.28 |
| CM | 7.61 | 405 | 60 | 21.4 | 602 | 70 | 9.13 |
|  | 7.59 | 390 | 80 | 22.1 | 760 | 110 | 9.42 |
|  | 7.58 | 493 | 70 | 25 | 882 | 95 | 9.28 |
| INF | 7.68 | 529 | 11 | 18.6 | 795 | 225 | 9.83 |
|  | 7.8 | 477 | 8 | 19.9 | 600 | 260 | 9.53 |
|  | 7.56 | 529 | 11 | 18.6 | 702 | 200 | 10.3 |
| RAS | 7.14 | 448 | 3.3 | 18.6 | 655 | 19740 | 11.0 |
|  | 7.62 | 492 | 5 | 18.7 | 482 | 20030 | 11.1 |
|  | 6.97 | 470 | 4.2 | 19.9 | 590 | 20700 | 11.2 |
|  |  |  |  |  |  |  |  |
| Samples | pH | Conductivity (μS/cm) | DO (% of air saturation) | Temperature (^o^C) | COD (mg/L or mg/gm) | DNA concentration (ng/mL) | Bacteria density log (cells /mL) |
| EFF | 7.68 | 493 | 14 | 19.6 | 86 | 70 | 8.74 |
|  | 7.28 | 473 | 17 | 19.2 | 26 | 110 | 8.72 |
|  | 7.61 | 514 | 12 | 19.7 | 90 | 95 | 8.78 |
| RD | 7.76 | 414 | 45 | 17.0 | 63 | 225 | 9.03 |
|  | 7.26 | 337 | 56.2 | 17.3 | 90 | 260 | 9.13 |
|  | 7.43 | 422 | 45 | 17.1 | 43 | 200 | 9.12 |
| SRD | 7.76 | 414 | 45 | 17.0 | 1610 | 26340 | 11.0 |
|  | 7.26 | 337 | 56.2 | 17.3 | 2500 | 20390 | 11.0 |
|  | 7.43 | 422 | 45 | 17.1 | 1760 | 24300 | 11.0 |

**Table S7A:** Data and statistical analysis of normalized data per bacterial cell for Class 1 integron (intI1). Normality was studied by the Shapiro-Wilk test; and the homoscedaticity of the variance was assessed using the Levene’s test. Data was not normal, therefore Krustall-Wallis test was performed to assess statistically significant differences, and Conover's-test was performed for pairwise comparison between sampling sites.

**Table S7B:** Data and statistical analysis of normalized data per bacterial cell for class 2 integron (intI2). Normality was studied by the Shapiro-Wilk test; and the homoscedaticity of the variance was assessed using the Levene’s test. Data was not normal, therefore Krustall-Wallis test was performed to assess statistically significant differences, and Conover's-test was performed for pairwise comparison between sampling sites.

**Table S7C:** Data and statistical analysis of normalized data per bacterial cell for class 3 integron (intI3). Normality was studied by the Shapiro-Wilk test; and the homoscedaticity of the variance was assessed using the Levene’s test. Data was not normal, therefore Krustall-Wallis test was performed to assess statistically significant differences, and Conover's-test was performed for pairwise comparison between sampling sites.

**Table S7D:** Data and statistical analysis of normalized data per bacterial cell for anthropogenic class 1 integron structures (aint1). Normality was studied by the Shapiro-Wilk test; and the homoscedaticity of the variance was assessed using the Levene’s test. Data was not normal, therefore Krustall-Wallis test was performed to assess statistically significant differences, and Conover's-test was performed for pairwise comparison between sampling sites.

**Table S7E:** Data and statistical analysis of normalized data per bacterial cell for empty class 1 integron structures (eaintI1). Normality was studied by the Shapiro-Wilk test; and the homoscedaticity of the variance was assessed using the Levene’s test. Data was not normal, therefore Krustall-Wallis test was performed to assess statistically significant differences, and Conover's-test was performed for pairwise comparison between sampling sites.

**Table S7F:** Data and statistical analysis of relative anthropogenic class 1 integron structures (aint1) per intI1 gene. Normality was studied by the Shapiro-Wilk test; and the homoscedaticity of the variance was assessed using the Levene’s test. Data was normal, therefore Anova test was performed to assess statistically significant differences, and Tukey contrasts-test was performed for pairwise comparison between sampling sites.

**Table S7G:** Data and statistical analysis of relative empty anthropogenic class 1 integron structures (eaint1) per anthropogenic class 1 integron structures (aint1). Normality was studied by the Shapiro-Wilk test; and the homoscedaticity of the variance was assessed using the Levene’s test. Data was normal, therefore Anova test was performed to assess statistically significant differences, and Tukey contrasts-test was performed for pairwise comparison between sampling sites.

**Table S8:** Diversity of bacteria families carrying integron class 1, 2, 3 or clinical class 1 integron. Results were summarised after search the NCBI_nr_ database against the conserved integron sequences (S_3.5). Dark grey represents the bacteria families that carries each integron gene, whereas X indicate the families found in at least one of the samples collected and analysed.


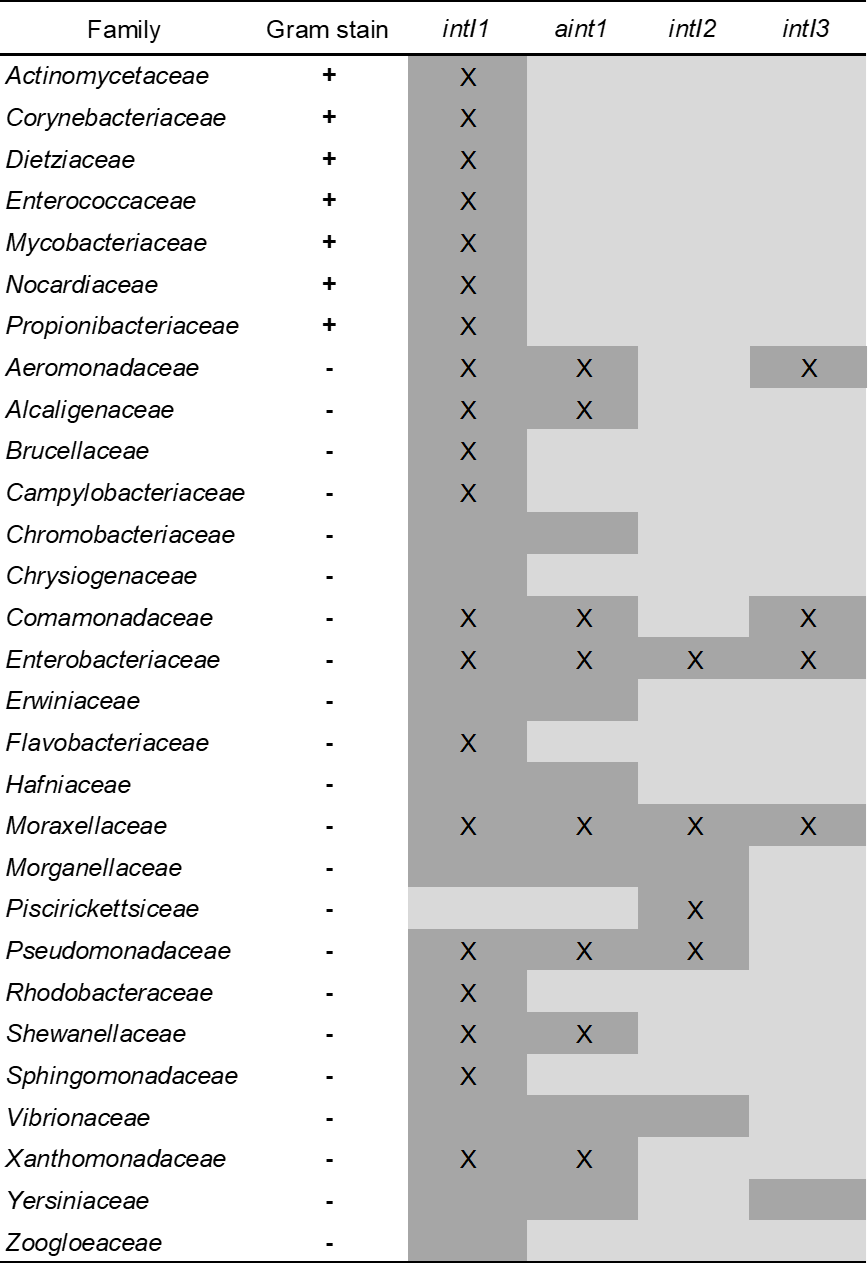


**Table S9:** Sample coverage. The percentage of total estimated species richness in a sample for the sample’s microbial community and the PBCMI.

| Site | Microbial community | | PBCMI | |
| --- | --- | --- | --- | --- |
| RU | 95.4 | ± 0.74 | 94.6 | ± 1.80 |
| SRU | 95.8 | ± 2.40 | 94.7 | ± 1.70 |
| CM | 98.8 | ± 0/16 | 97.7 | ± 0.13 |
| HP | 98.5 | ± 0.84 | 97.8 | ± 0.29 |
| INF | 97.4 | ± 0.17 | 98.1 | ± 0.45 |
| RAS | 99.0 | ± 0.33 | 97.5 | ± 0.19 |
| EFF | 96.1 | ± 0.58 | 97.4 | ± 0.28 |
| RD | 96.9 | ± 1.30 | 97.1 | ± 0.68 |
| SRD | 96.6 | ± 1.60 | 94.4 | ± 1.20 |

**Figure S1:** Multiple sequence alignment tree of the integrase from the clinical class 1 integron (*clintI1*) and the pre-clinical class 1 integron (*pclintI1*) structures. We define pre-clinical class 1 integrons are the evolutionary pre-cursor to *clintI1*. Clustering shows that the integrase gene do not discriminate between *clintI1* and *pclintI1* structures.


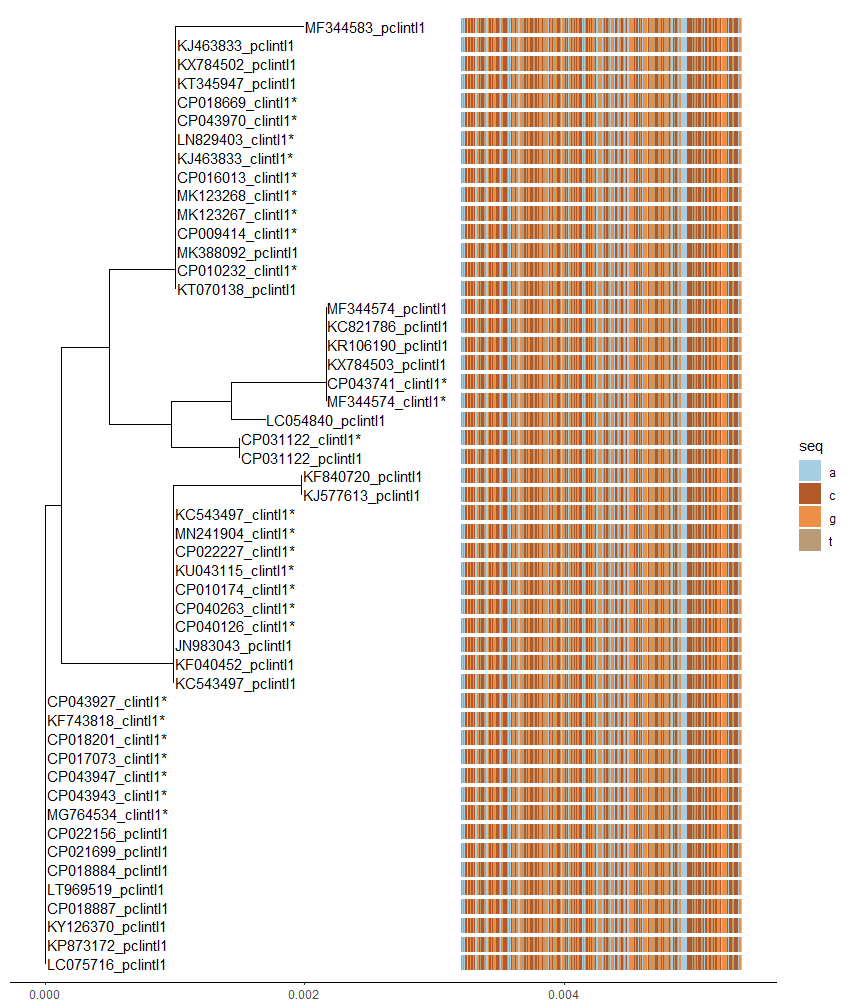


**Figure S2A:** Conserved region from the anthropogenic class 1 integron structure (i.e., qacEΔ1/sul1 region). Sequences were aligned in Clustal X 2.0, and primers and TaqMan probe were designed in this region (set B in Figure 3).


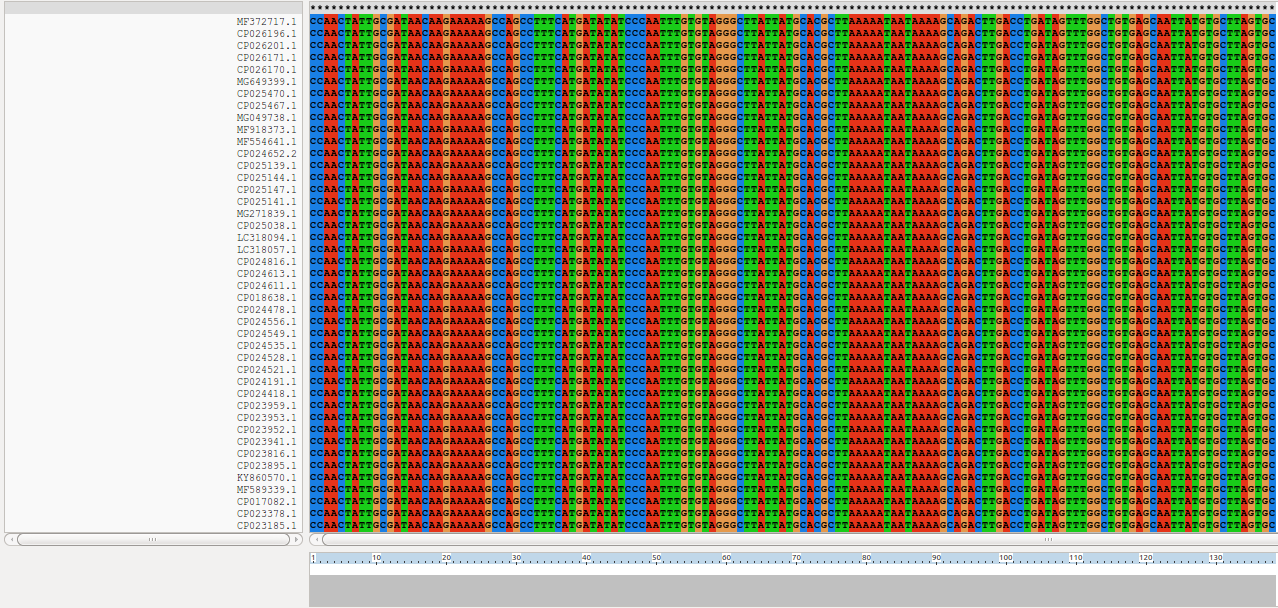


**Figure S2B:** Conserved region from the empty anthropogenic class 1 integron structure. Sequences were aligned in Clustal X 2.0, and primers and TaqMan probe were designed in this region (set C in Figure 2).


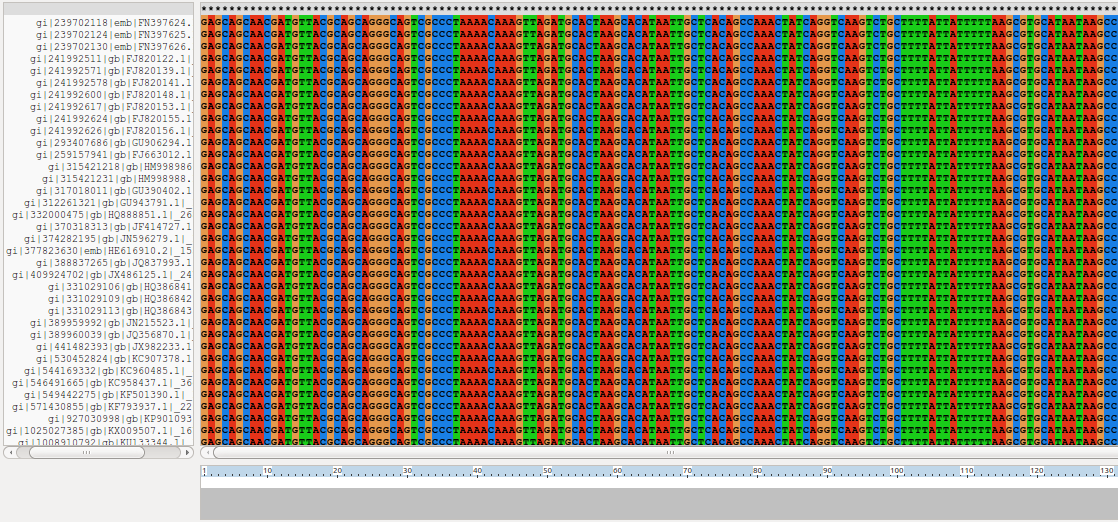


**
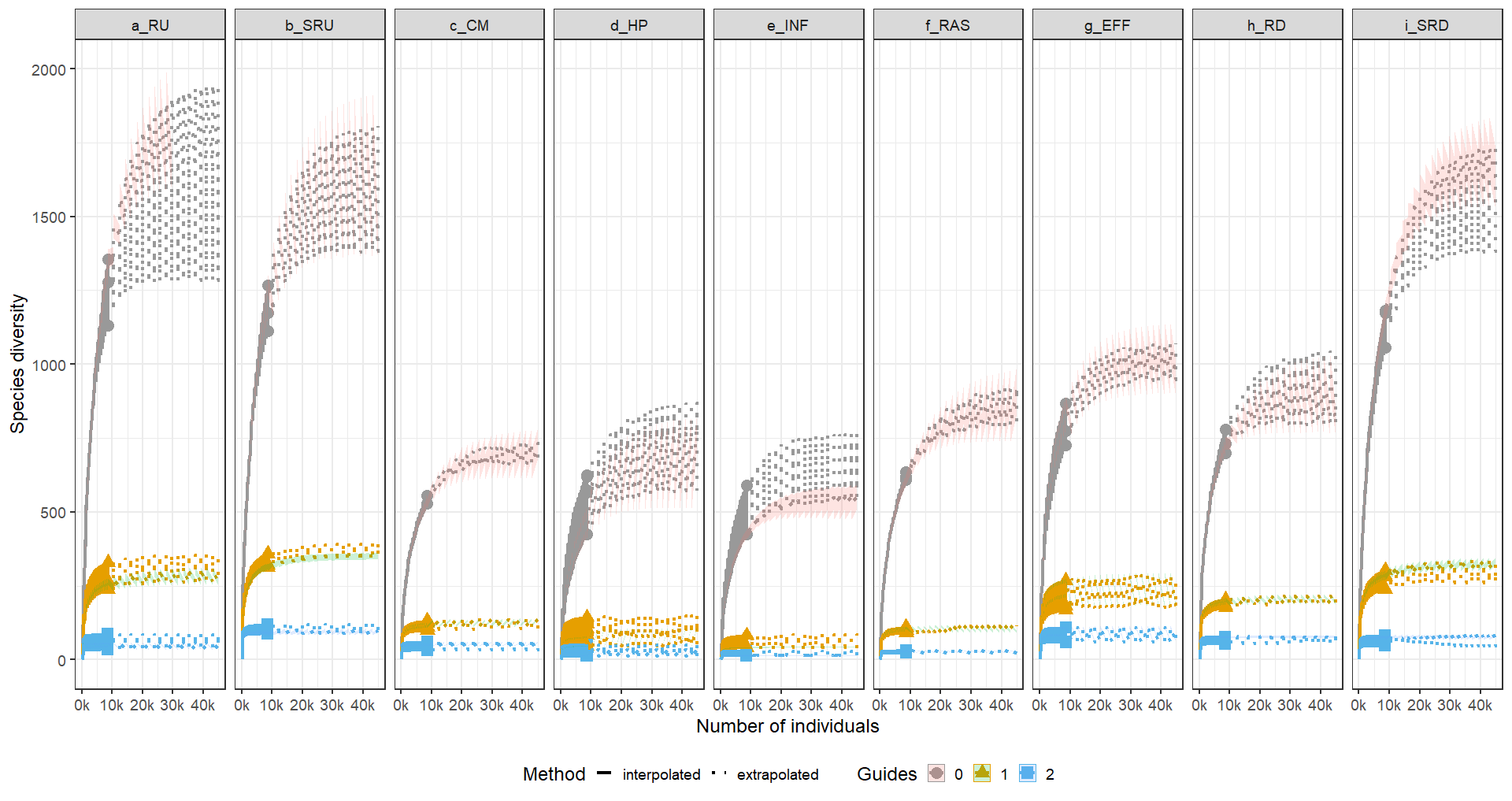
Figure S****3:** Diversity profile curves of microbial communities, using plots of Hill numbers q D(') as a function of order q, 0 q 3. In this study, we used the first three Hill numbers to characterise the diversity of a species assemblage based on the individual-based data: species richness (q = 0), the exponential of the Shannon entropy (Shannon diversity, q = 1), and the inverse Simpson concentration (Simpson diversity, q = 2). The proposed estimators are accurate for both rarefaction (solid line) and short-range extrapolation (dashed line).

**Figure S4:** Diversity profile curves of PBCMI, which plots Hill numbers q D(') as a function of order q, 0 q 3. The first three Hill numbers were used to characterise the diversity of a species assemblage based on the individual-based data: species richness (q = 0), the exponential of the Shannon entropy (Shannon diversity, q = 1), and the inverse Simpson concentration (Simpson diversity, q = 2). The proposed estimators are accurate for both rarefaction (solid line) and short-range extrapolation (dashed line).


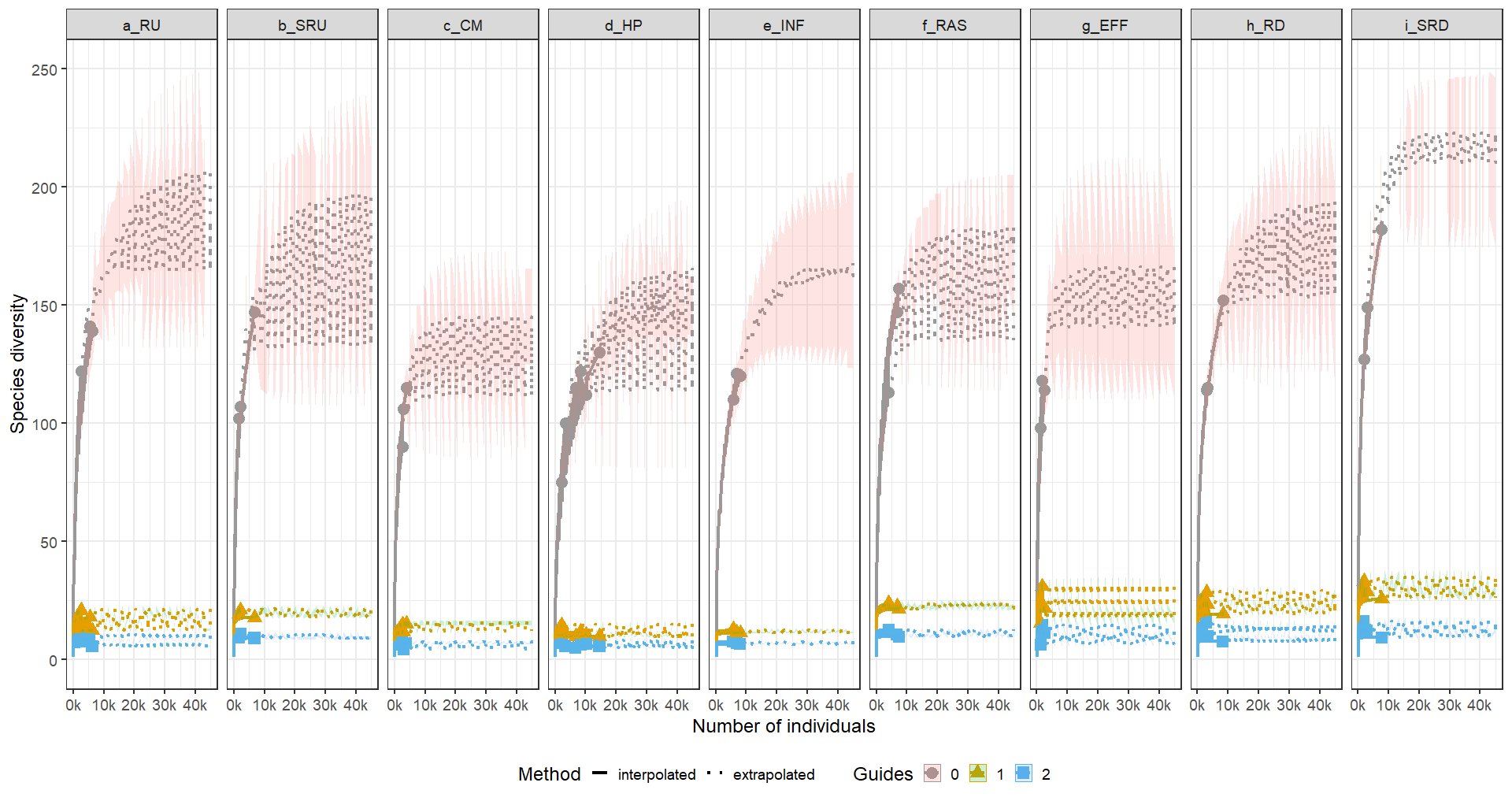


**Figure S5:** Cluster dendrograms of community composition dissimilarity (Bray-Curtis, average neighbour clustering) based on A) OTU distributions (all OTUs), and B) OTUs only from PBCMI families among sampling sites. The dendograms show four clusters at 0.72 cut-off (microbial community) and 0.52 (PBCM): Clusters comprise: CM, HP and INF, i.e., raw wastewater; EFF and RD, i.e., effluent and downstream water column, and RAS and SRD, i.e., recycle solids and downstream sediments; RU and SRU, i.e., upstream river sites.


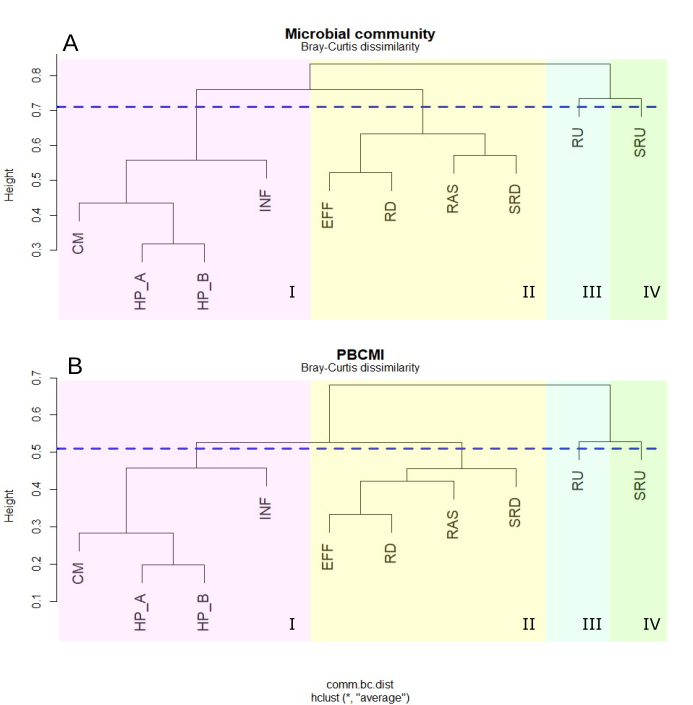


**Figure S6:** Heatmap and cluster analysis (Bray–Curtis; complete linkage) of PBCMI (OUT level) for different sampling points. Relative abundances are log-transformed for visualization purposes. PBCMI (x-axis) and samples (y-axis) are clustered based on similar abundance and occurrence patterns. Intensity of colours indicate relative abundance gradient of the PBCMI at each point (red = high abundance and pale-yellow = no presence). Sample codes: SRU) upstream sediments, CM) community wastewater, HP) hospital wastewater, INF) WWTP Influent, RAS) recycled activated sludge, EFF) WWTP effluent, RD) downstream water column, and SRD) downstream sediments.

*
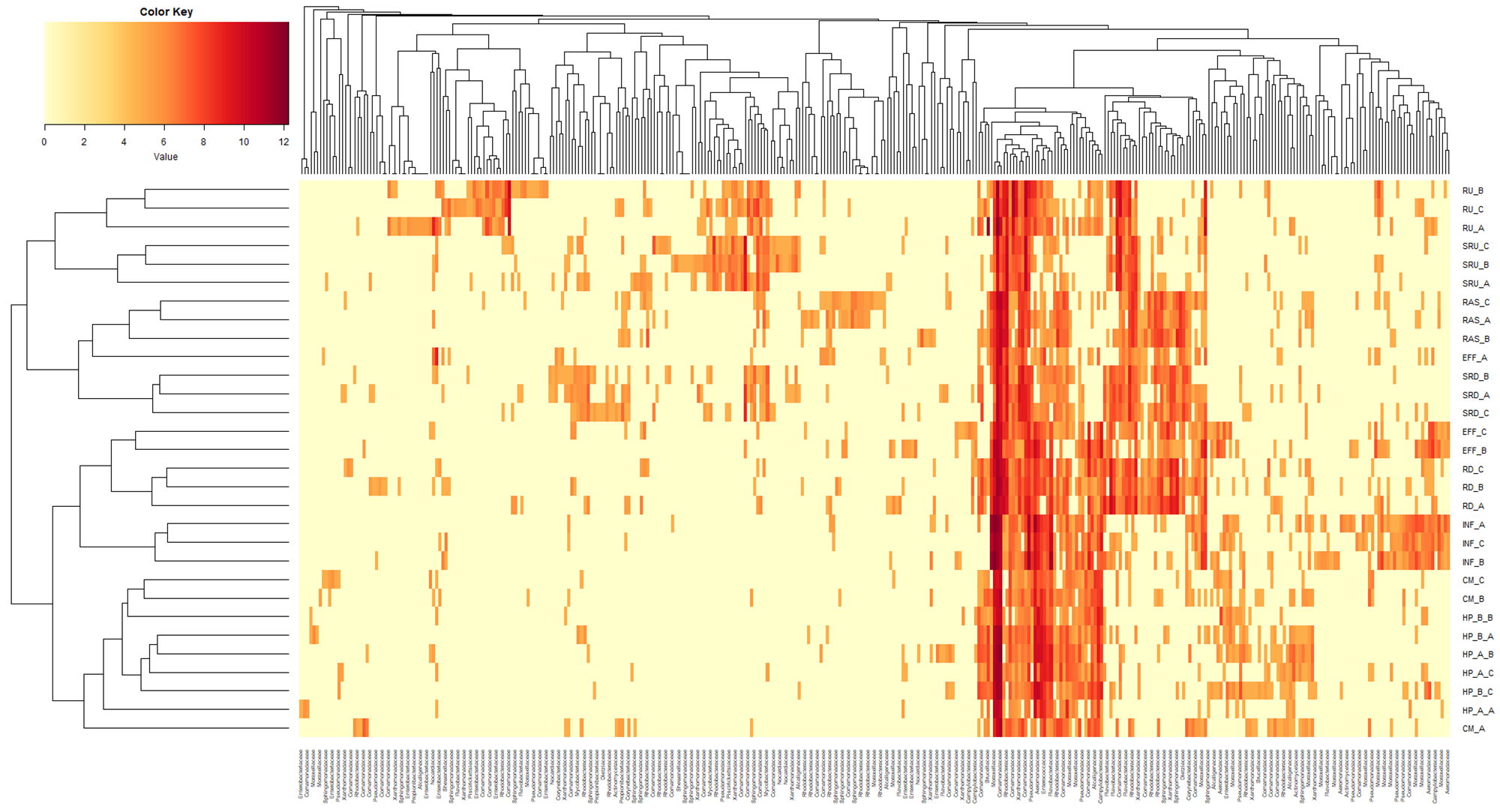
*

**Figure S7:** A linear discriminant analysis effect size (LEfSe) method identifies the significantly different abundant taxa of bacteria in all the sampling sites (family level). Each bacteria family has an effect size LDA score. Only taxa meeting an LDA significance threshold of >2 are shown. Samples are grouped by sampling site: CM) community sewage, HP) hospital sewage, INF) WWTP influent STP, EFF) WWTP effluent, RAS) recycled activated sludge, RU) upstream water column, SRU) upstream sediments, RD) downstream water column, and SRD) downstream sediments.


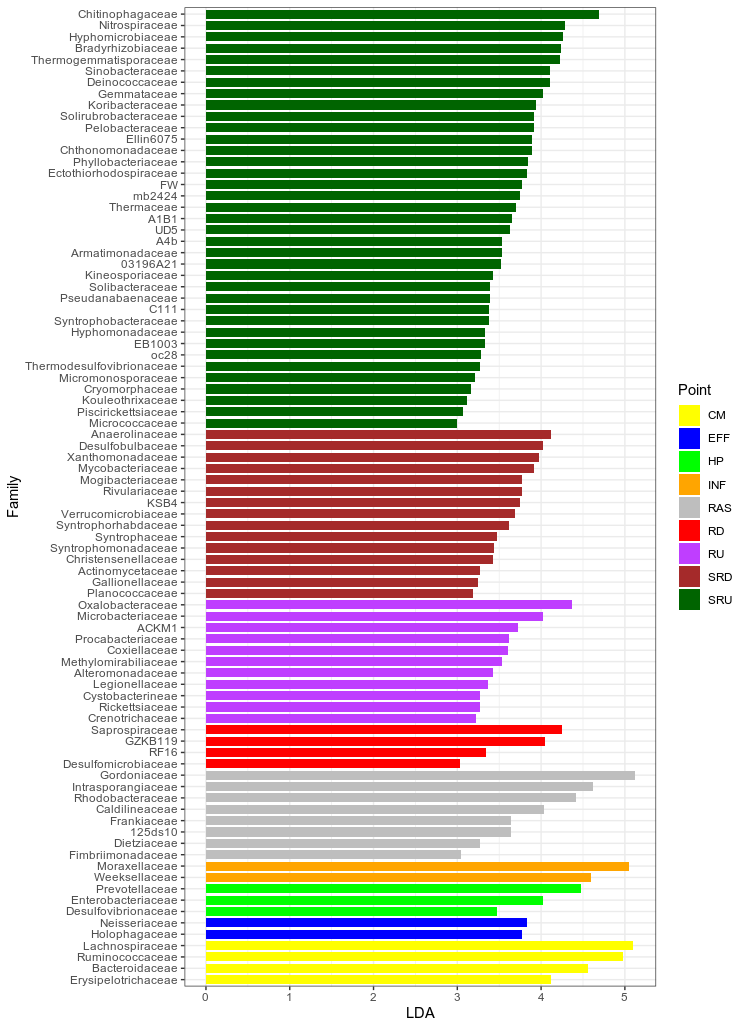


**Figure S8:** A network analysis revealing the co-occurrence bacteria taxa (family level) and mobile integrons (MIs). Connections represent a Spearman’s correlation coefficient p > 0.4 and significant correlation (p-value > 0.05). The PBCMI are highlighted in red text. The network shows the presence of four different modules, which are highlighted in different colours. Module I primarily represent bacteria from raw wastewater sources (CM, HP, INF), module II represents bacteria from the WWTP or the downstream water column (RAS, EFF, RD, SRD). Taxa in Module III are from the upstream water column (RU) and Module IV river sediments (SRU).
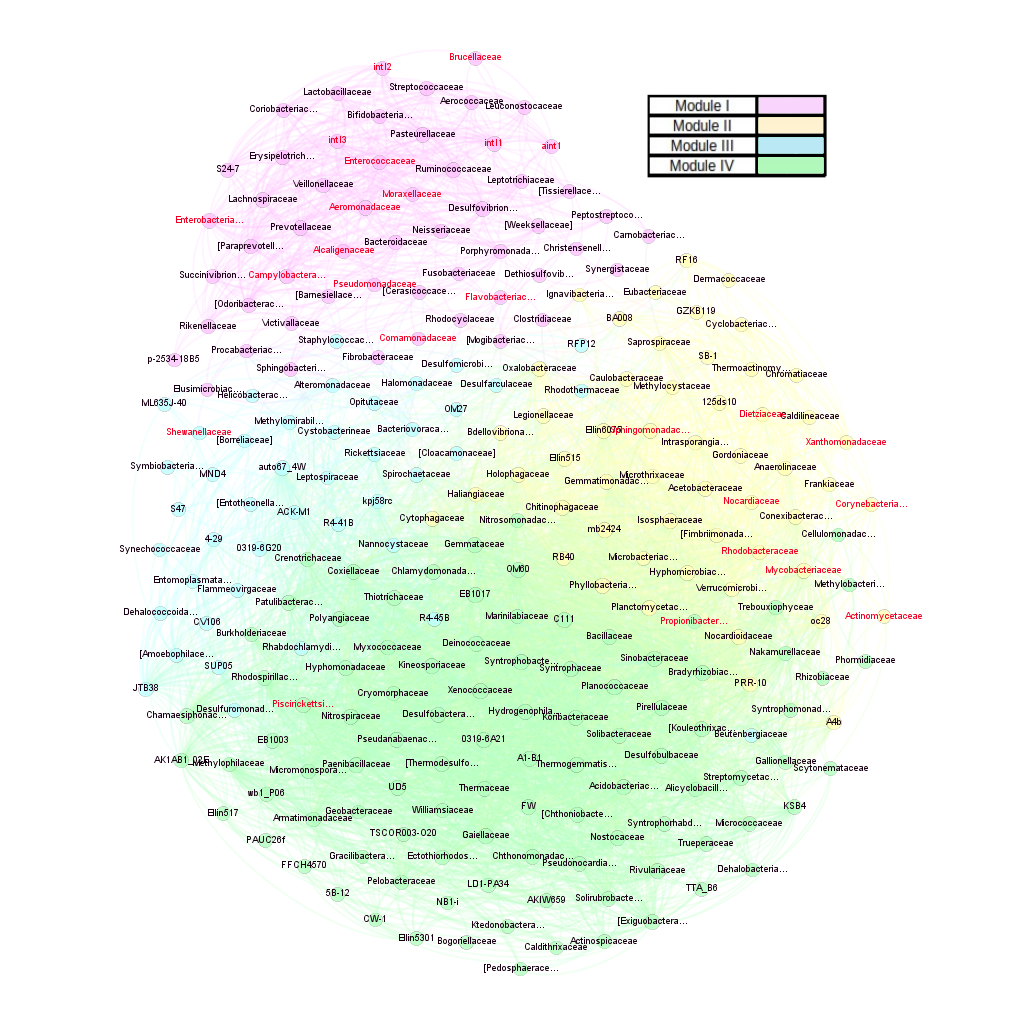


**Figure S9A:** Module I represent mostly bacteria from the wastewater sources (i.e., CM, HP, INF) and contain all the detected MIs. Bacterial families that most likely explain differences between water network compartments are differentiated by colour. PBCMI are highlighted in red text. Bacteria that do not clearly link to water network compartment are in pale yellow.


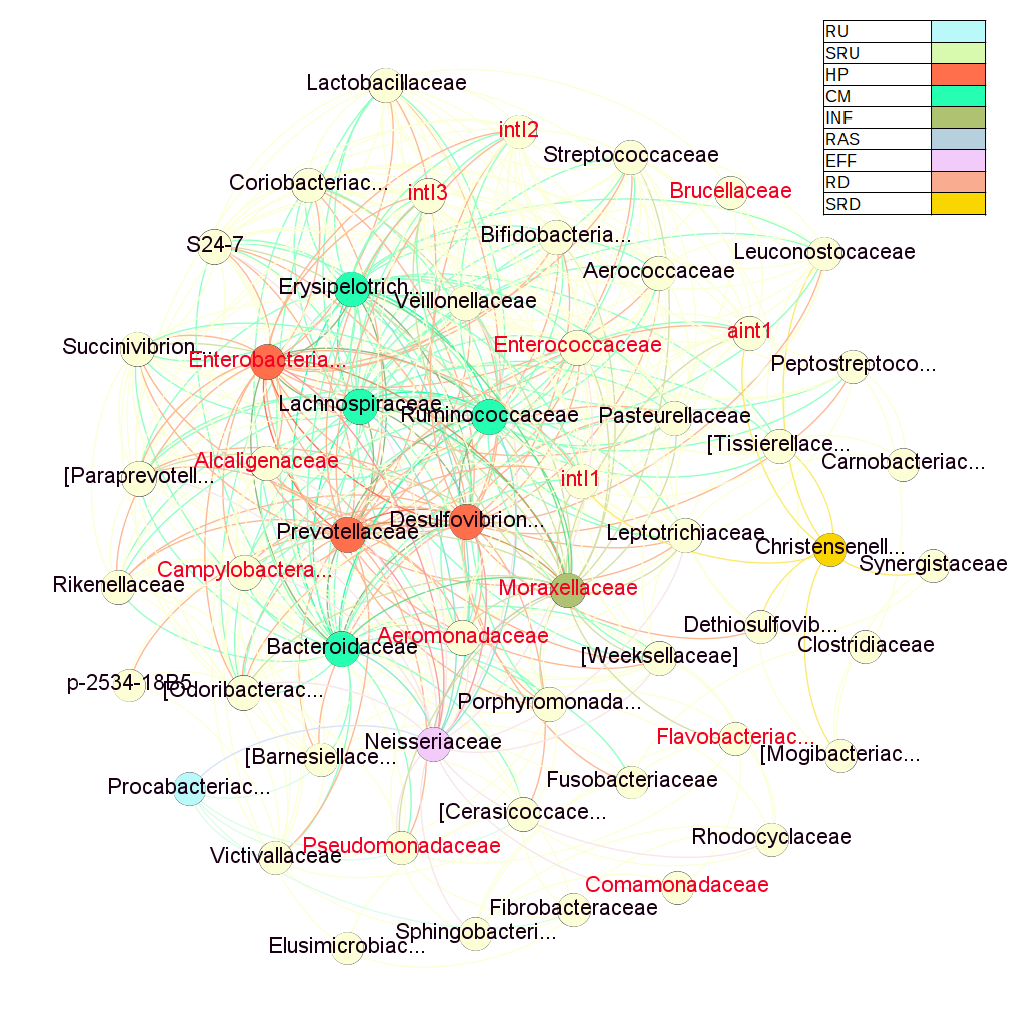


**Figure S9B:** Module II primarily represents bacteria from human-impacted environments (RAS, EFF, RD, and SRD). Bacterial families that most likely explain differences between water network compartments are differentiated by colour. PBCMI are highlighted in red text. Bacteria that do not clearly link to water network compartment are in pale yellow.

**Figure S9C:** Module III represents mostly bacteria from the water column from the river upstream (RU). Bacterial families that most likely explain differences between water network compartments are differentiated by colour. PBCMI are highlighted in red text. Bacteria that do not clearly link to water network compartment are in pale yellow.
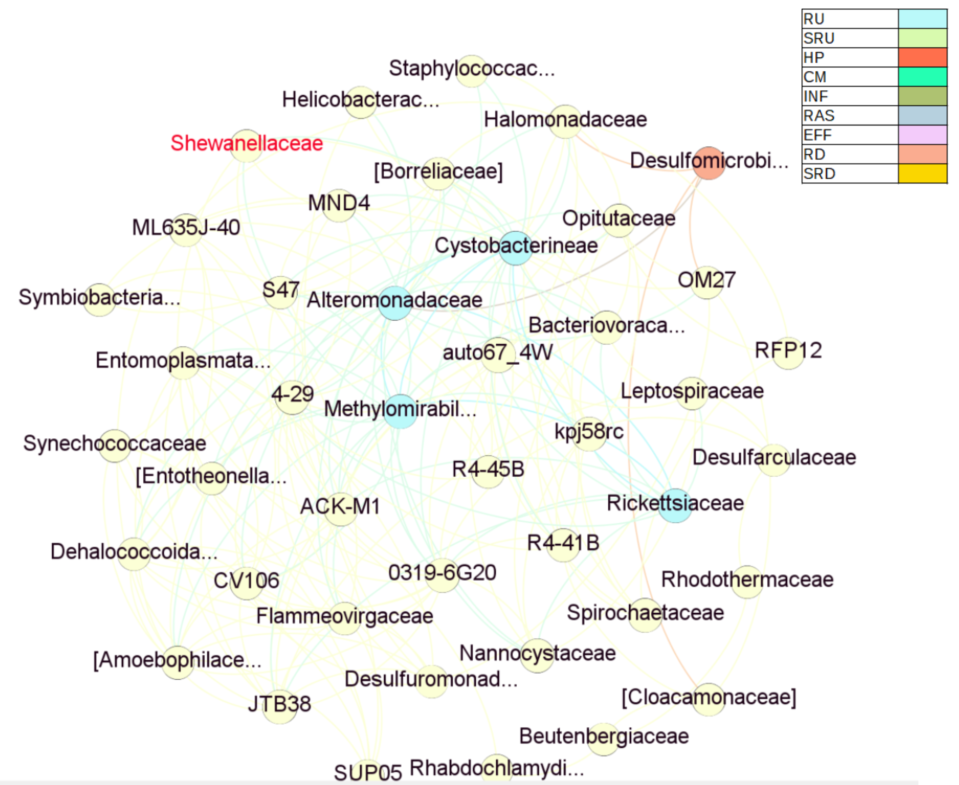


**Figure S9D:** Module IV represents mostly bacteria from non-human environmental bacteria (SRU). Bacterial families that most likely explain differences between water network compartments are differentiated by colour. PBCMI are highlighted in red text. Bacteria that do not clearly link to water network compartment are in pale yellow.

**References**

Barraud, O, Baclet, C., Denis, F., Ploy MC. Quantitative multiplex real-time PCR for detecting class 1, 2 and 3 integrons. (2010). J Antimicrob Chemother. 65(8), 1642–1645.

Edgar RC. (2004). MUSCLE: multiple sequence alignment with high accuracy and high throughput. Nucleic Acids Res. 32(5), 1792–1797.

Gillings MR, Duan X, Hardwick SA, Holley MP, Stokes HW. (2009). Gene Ccssettes encoding resistance to quaternary ammonium compounds: A role in the origin of clinical class 1 integrons?” ISME J. 3(2), 209–15.

Gillings MR, Gaze WH, Pruden A, Smalla K, Tiedje JM, Zhu YG. (2015). Using the class 1 integron-integrase gene as a proxy for anthropogenic pollution. ISME J. 9(6), 1269-1279.

Holmes AJ, Gillings MR, Nield BS, Mabbutt BC, Nevalainen KH, Stokes HW. (2003). The gene cassette metagenome is a basic resource for bacterial genome evolution. Environ Microbiol. 5(5), 383-394.

Lévesque C, Piché L, Larose C, Roy PH. (1995). PCR mapping of integrons reveals several novel combinations of resistance genes. Antimicrob Agents Chemother. 39(1), 185-91.

Marquez C, Labbate M, Raymondo C, Fernandez J, Gestal AM, Holley M, *et al.* (2008). Urinary tract infections in a South American population: dynamic spread of class 1 integrons and multidrug resistance by homologous and site-specific recombination. J Clin Microbiol. 46, 3417–3425.

White PA, McIver CJ, Rawlinson WD. (2001). Integrons and gene cassettes in the Enterobacteriaceae. Antimicrob Agents Chemother. 45(9), 2658–2661.

Yu G, Smith DK, Zhu H, Guan Y, Lam TTY. (2017). ggtree: an R package for visualization and annotation of phylogenetic trees with their covariates and other associated data. *Methods Ecol. Evol.* 8, 28-36.
